# Novel Optoelectronic Reconfigurable Transistors Based on Graphene/VO_2_ Heterojunction for Efficient Neuromorphic Perception, Computation, and Storage

**DOI:** 10.1002/advs.202513429

**Published:** 2025-09-26

**Authors:** Danke Chen, Yuning Li, Xiaoqiu Tang, Jingye Sun, Xuan Yao, Peizhi Yu, Xue Li, Qing You, Hanyu Wang, He Tian, Tao Deng

**Affiliations:** ^1^ School of Electronic and Information Engineering Beijing Jiaotong University Beijing 100044 P. R. China; ^2^ Department of Precision Instrument Tsinghua University Beijing 100049 P. R. China; ^3^ School of Integrated Circuits and Beijing National Research Center for Information Science and Technology (BNRist) Tsinghua University Beijing 100049 P. R. China

**Keywords:** low‐dimensional materials, neuromorphic devices, optoelectronics, reconfigurability

## Abstract

Optoelectronic artificial neuromorphic devices, inspired by biological vision systems, have overcome bottlenecks of the von Neumann architecture. The innovation and integration of neuromorphic hardware systems represent a pivotal challenge for advancing the iteration of artificial intelligence. Accordingly, a novel optoelectronic reconfigurable neuromorphic transistor (ORNT) is designed to integrate three functions, enabling the perception, computation, and storage of optical information in a manner analogous to visual nervous systems. Based on the electrode‐inserted graphene/VO_2_ nanoparticles heterostructure and photovoltaic effect, the ORNT demonstrates broadband self‐powered responsiveness from the ultraviolet to near‐infrared (365–940 nm). Leveraging the photogating effect and the photoinduced phase transition in VO_2_, the differentiated electrode design enables wide‐electrode ORNTs to exhibit synaptic behavior under bias voltages, whereas narrow‐electrode ORNTs demonstrate data storage capability and multistage photomodulation. Furthermore, an integrated optical communication and processing‐in‐memory system is developed, achieving a full‐process demonstration from optical perception to computation and storage. Overall, the ORNTs introduced in this work provide an innovative strategy for optimizing the hardware resource allocation of chips and enhancing the adaptability and scalability of systems.

## Graphical abstract

A novel optoelectronic reconfigurable neuromorphic transistor based on a graphene/VO_2_ heterojunction with inserted Au electrodes is proposed. It exhibits broad‐spectrum self‐powered photodetection capability ranging from 365 to 940 nm. Through bias control and electrode sizing strategies, multiple functions for switch, synapse, and storage are achieved, enabling faithful emulation of the complete visual neural process.

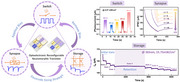

## 1 Introduction

In the era of artificial intelligence (AI), the explosive growth of data has exposed the limitations of the von Neumann architecture in terms of computational latency and excessive power consumption.^[^ ^1^ ^]^ Coupled with the fact that traditional silicon‐based CMOS devices are approaching fundamental scaling limits, Moore's Law is becoming less effective in guiding the innovation of next‐generation chips.^[^ ^2^, ^3^, ^4^ ^]^ Researchers have begun to draw on primordial life activities and electronics theories to construct hardware systems based on neuromorphic devices.^[^ ^5^, ^6^ ^]^ Bio‐inspired neuromorphic devices could mimic numerous key nodes of biological neural systems to acquire information. Representative devices such as receptors,^[^ ^7^, ^8^, ^9^ ^]^ artificial synapses,^[^ ^10^, ^11^, ^12^ ^]^ artificial neurons,^[^ ^13^, ^14^, ^15^ ^]^ memories,^[^ ^16^, ^17^, ^18^ ^]^ filters^[^ ^19^, ^20^ ^]^ have shown excellent perception, integration, memory, and recognition capabilities. However, most neuromorphic devices are limited by specific executions and discrete applications, lacking all‐in‐one multifunctional integration strategies to fulfill adaptive and scalable requirements. Currently, energy‐intensive and area‐inefficient hardware systems prompt the development of integrating multiple advanced neural functions in one manner. Reconfigurable neuromorphic devices refer to bioinspired hardware components that emulate neural behaviors and dynamically adapt their functionalities. This adaptation occurs within the same device and is driven by operational demands.^[^ ^21^ ^]^ Reconfigurable neuromorphic devices eliminate functional constraints among disparate components by utilizing identical structures and materials to circumvent heterogeneous process incompatibilities, and adaptively allocate hardware resources for energy‐efficient and high‐performance operation.^[^ ^22^, ^23^ ^]^ Several studies have demonstrated functional switching through diverse reconfiguration strategies: i) Structural designs, such as ionic conductive layers,^[^ ^24^, ^25^ ^]^ semi‐floating gates,^[^ ^26^ ^]^ heterojunctions,^[^ ^27^ ^]^ and multiple electrodes;^[^ ^28^, ^29^ ^]^ ii) Material applications, including polarization,^[^ ^30^, ^31^, ^32^ ^]^ doping,^[^ ^33^, ^34^, ^35^ ^]^ phase transition,^[^ ^36^, ^37^ ^]^ defect engineering,^[^ ^38^ ^]^ and conductive pathway modulation.^[^ ^39^, ^40^, ^41^ ^]^ Nevertheless, they face two fundamental limitations: i) Structural complexity: most designs require intricate architectures and fabrication processes to achieve multifunctional reconfigurations; ii) Unimodal control: the focus remains predominantly on electrical modulation and neglects natural stimuli (e.g., optical and mechanical inputs), which is critical for neuromorphic‐environment interactions. A comparison of reconfigurable neuromorphic devices is listed in Table S1 (Supporting Information).

**Figure 1:**
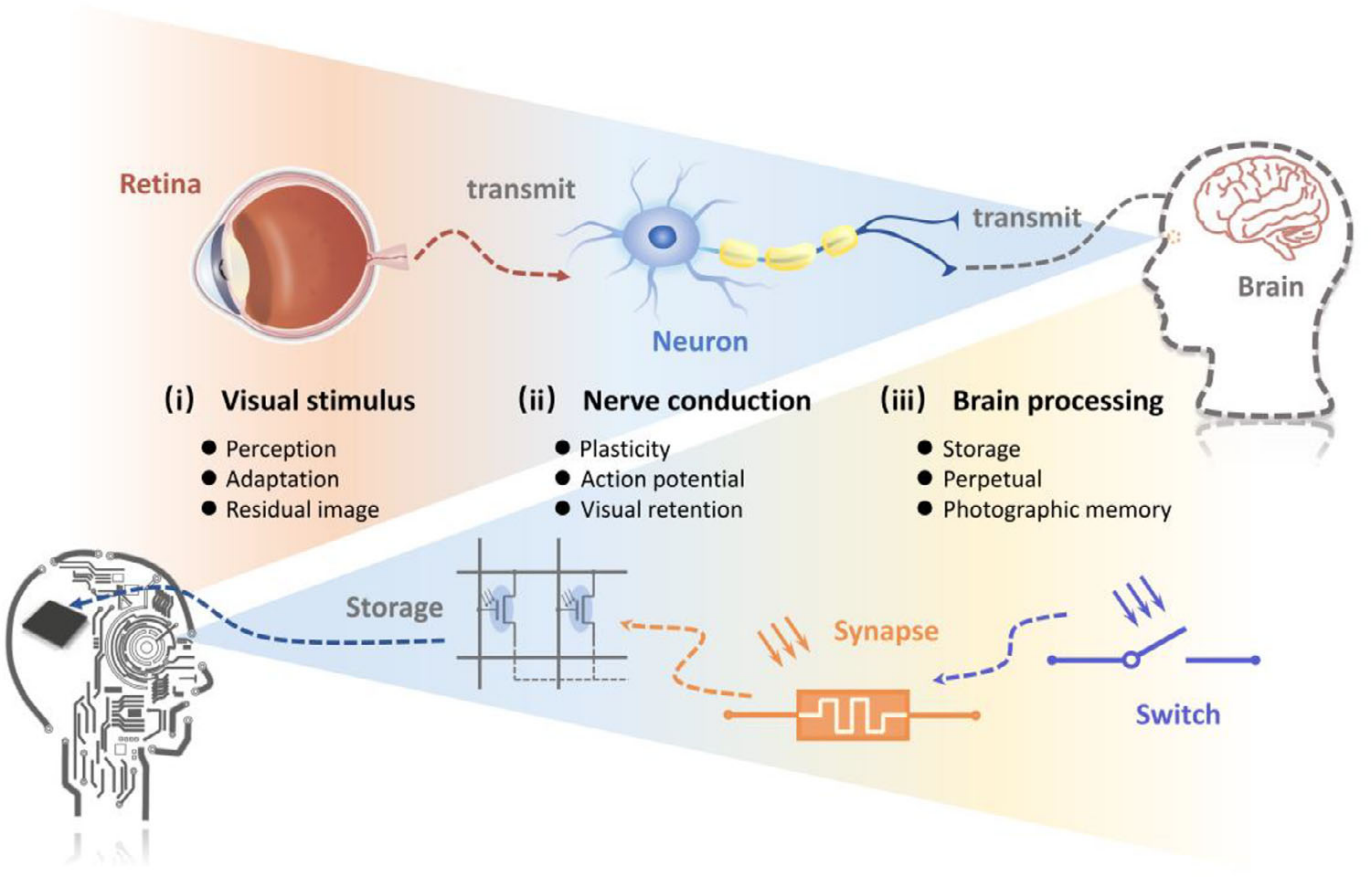
Schematic of a biological visual system and an artificial optoelectronic neuromorphic system.

It is well‐established that light information exchange with the surroundings is a key biological trait. Retinal photoreceptors mediate light detection and adaptation, with subsequent transmission of neural impulses via the optic nerve to the occipital cortex for visual processing. Behaviorally salient or repeatedly processed visual information is then selectively routed to the frontal and hippocampal regions for long‐term memory consolidation. Notably, emerging evidence demonstrates that non‐invasive light stimulation can directly modulate the cortical activity to enhance the visual working memory performance.^[^ ^42^ ^]^ Photonic signals exert both direct and indirect effects across hierarchical stages of the visual system. The artificial neuromorphic optoelectronic system emulates the core stages of biological visual processing by integrating three representative functional components. The optoelectronic switches exhibit rapid response to optical stimuli, with magnitude modulated by both wavelength and intensity of the incident light. The artificial optoelectronic synapses modulate the intensity and temporal characteristics of signal transmission through a tunable weighting mechanism. The storage elements maintain a defined state upon receiving repeated or high‐intensity stimuli, persisting until the next arrival of stimulus. The correspondence between the biological visual neural system and the artificial optoelectronic neuromorphic system is shown in **Figure** **1**.

Owing to the device characteristics for the artificial optoelectronic neuromorphic system and the bottlenecks of efficient integration, we herein propose a novel optoelectronic reconfigurable neuromorphic transistor (ORNT) based on a gold (Au)‐electrode‐inserted graphene/VO_2_ nanoparticles (NPs) heterostructure. The ORNTs achieve self‐powered broadband switching responses spanning from the ultraviolet to near ‐ infrared (UV‐NIR, 365‐940 nm). It has been demonstrated that an excellent photoresponsivity of 3.46 A W^−1^ and a high sensitivity of 2.50×10^9^ Jones (1 Jones = 1 cm Hz^1/2^ W^−1^) were obtained with 940 nm illumination. Both the rise time (*t*_r_) and decay time (*t*_d_) were on the order of ≈30 ms. Under an applied bias voltage, the electrode size strategy of ORNTs enables the faithful simulation of visual information computation and storage. The wide‐electrode ORNTs demonstrate synaptic plasticity, thereby achieving the transition from short‐term memory (STM) to long‐term memory (LTM) under persistent and repetitive optical stimuli. Such plasticity is also realized in electrical synaptic simulations, and the optoelectronic co‐activation facilitated reversible and controllable synaptic weight modulation. The narrow‐electrode ORNTs exhibit nonvolatile data storage with a retention time exceeding 10 years, alongside optically programmable multi‐level states. Moreover, a system‐level verification of infrared communication and ultraviolet information processing was proposed to demonstrate the application value of the ORNT. Overall, this work provides a novel optoelectronic strategy that achieves reconfigurable switch‐synapse or switch‐storage functions in a single device, thereby offering a promising pathway to optimize the hardware resource allocation and enable more flexible and efficient AI.

## 2 Results

### 2.1 Structure and Characterization of the ORNT

**Figure 2:**
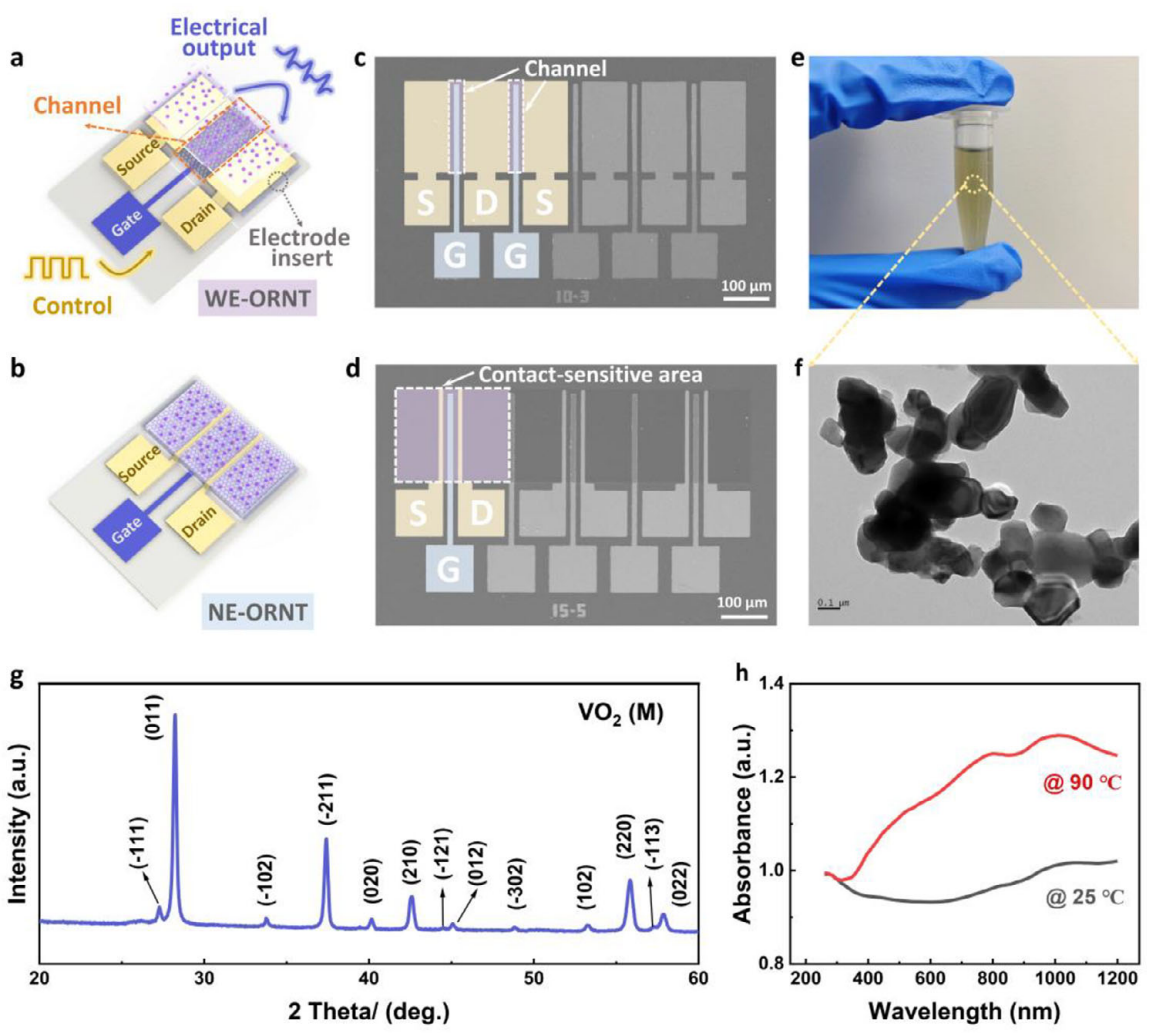
The structure and characterization of the ORNT. a‐b) Structures of the WE‐ORNT and NE‐ORNT consisting of Au‐electrode‐inserted graphene/VO_2_ NPs heterostructure. c,d) Top‐view SEM image of five parallel WE‐ORNTs and NE‐ORNTs. e) A photograph of the as‐prepared VO_2_ NPs ethanol solution. f) High‐resolution transmission electron microscopy (TEM) characterization of dispersed VO_2_ nanoparticles. g) XRD pattern of VO_2_ NPs with reference peaks for monoclinic (M) phase (JCPDS # 82–0661).^[^ ^43^ ^]^ h) Absorption spectra (260–1200 nm) of VO_2_ NPs at 25 °C (black line) and 90 °C (red line).

The optoelectronic reconfigurable device reported in this paper exhibits a three‐terminal field‐effect transistor architecture. As schematically illustrated in **Figure** **2**a,b, two distinct electrode engineering strategies were employed in the ORNTs, differentiated solely by subtle modifications to the electrode dimensions. The source‐drain electrode widths were designed at 100 µm for wide‐electrode ORNT (WE‐ORNT) and 10 µm for narrow‐electrode ORNT (NE‐ORNT), with the channel dimensions maintained constant at 30 µm in width and 200 µm in length. The functional region of the ORNT comprises vertically stacked heterojunctions of monolayer graphene/VO_2_ NPs interleaved with Au electrodes. Strategically placed electrodes between graphene and VO_2_ layers serve to enhance the extraction efficiency of photogenerated carriers. Buried‐gate design combines low‐voltage operation with wide‐area photosensitivity, overcoming the limitations of back‐gate architecture. Figure 2c,d displays the scanning electron microscopy (SEM) top‐view image of five parallel WE‐ORNTs and NE‐ORNTs. The large‐area‐grown monolayer graphene was intact transferred onto the SiO_2_ dielectric layer, and patterned. VO_2_ NPs (100–300 nm, as shown in Figure 2e,f) were dispersed in ethanol after grinding and centrifugation, and then uniformly coated on the device surface. The experimental section provides a full description of the fabrication procedure. X‐ray diffraction (XRD) analysis in Figure 2g confirmed the crystalline properties of VO_2_ NPs. The dominant diffraction peaks were located at 28.24°, 37.42°, 42.56°, and 55.82°, which were assigned to the monoclinic crystalline phase (space group: *P*2_1_/*c*) of VO_2_ (M).^[^ ^43^, ^44^ ^]^ Figure 2h presents the temperature‐dependent absorption spectra of VO_2_ NPs. Owing to their intrinsic material properties and the small size effect, VO_2_ NPs exhibit prominent absorption peaks in the UV and NIR regions, acting as the primary photosensitive layer that governs the overall photoresponse of the device. The NIR absorption intensity increases markedly upon heating due to the phase transition of VO_2_ from an insulating monoclinic phase to a metallic rutile structure (Figure 2h, red line). All experiments described below were conducted under ambient conditions (25 °C).

### 2.2 Optoelectronic Switching Behaviors of the ORNT

**Figure 3:**
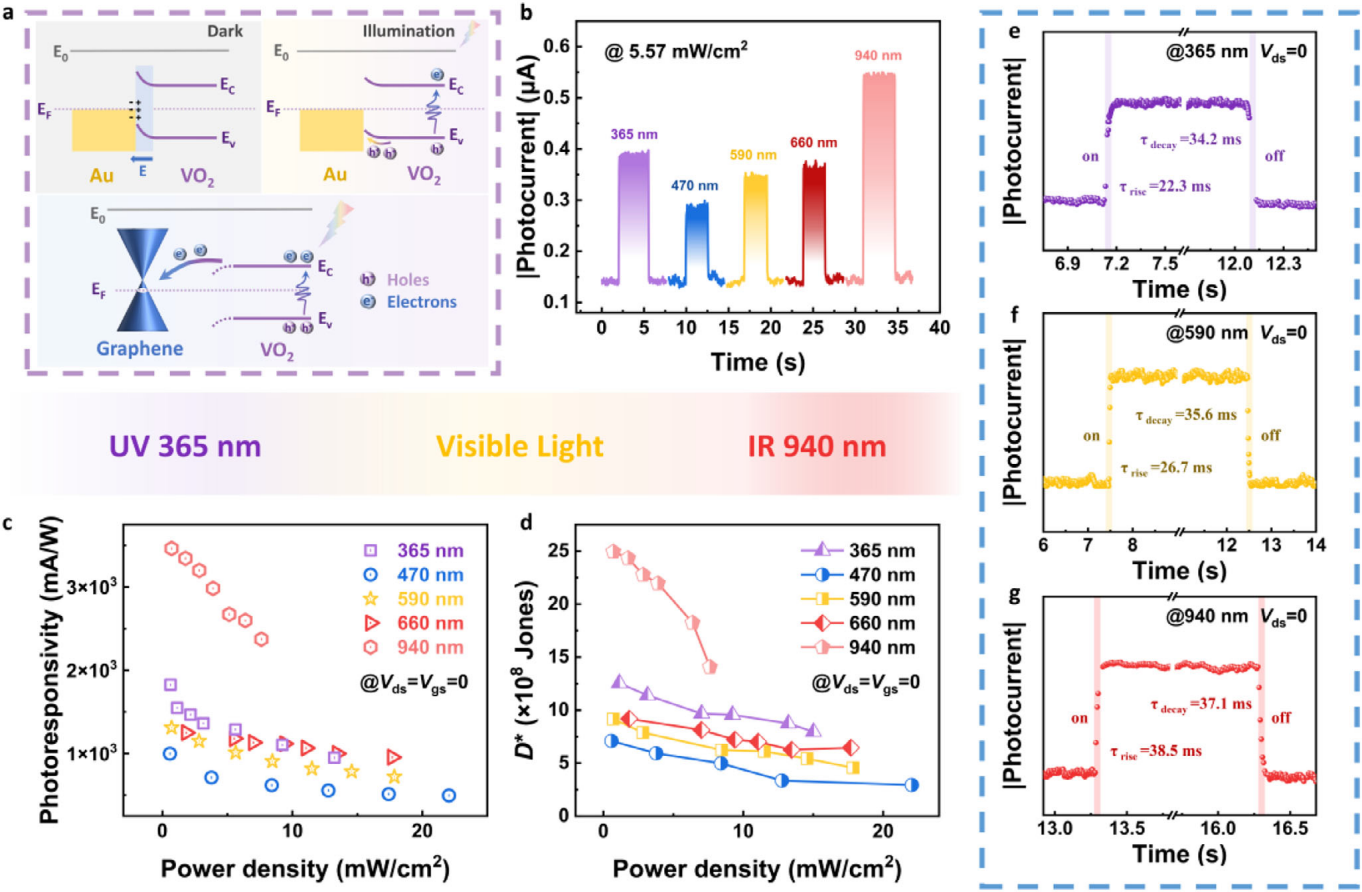
Optoelectronic switching characteristics of the WE‐ORNT. a) Schematic diagram of the band structures of the WE‐ORNT and the tendency of charge transfer. b) Photocurrent responses of the WE‐ORNT across the UV–NIR spectra under the same (5.57 mW cm^−2^) illumination power density. c,d) *R* and D* for different wavelengths at *V*_ds_ = *V*_gs_ = 0 V under various power densities. e‐g) The time‐resolved photoresponses of the WE‐ORNT at 365, 590, and 940 nm.

Within this section, the optoelectronic properties of the WE‐ORNT were initially tested under an unbiased voltage state (source–drain voltage, *V*_ds_ = 0; gate voltage, *V*_gs_ = 0). Figure S1 (Supporting Information) shows rapid rise/decay dynamics in both monolayer graphene and graphene/VO_2_ NPs heterostructure devices under 940 nm laser irradiation, consistent with a self‐powered switching capability. The heterostructure device exhibited a significantly enhanced normalized photocurrent response compared to its monolayer counterpart. To clearly understand the working mechanism of WE‐ORNTs, the electrical transfer characteristics of monolayer graphene and graphene/VO_2_ heterojunction devices were examined first. As depicted in Figure S2a (Supporting Information), the monolayer graphene transistor exhibits p‐type doping, arising from defects and surface adsorbates introduced during the fabrication and transfer processes.^[^ ^45^, ^46^ ^]^ The incorporation of VO_2_ NPs also induces additional doping effects, as supported by previous studies (Figure S2b, Supporting Information).^[^ ^47^ ^]^ These unavoidable defects and surface adsorbates also induce a small contact barrier between the graphene and the gold electrode.^[^ ^48^, ^49^ ^]^ In pure monolayer graphene devices, photoexcitation generates limited electron‐hole pairs. These carriers are effectively separated by the built‐in potential and collected through external circuits, resulting in a small photocurrent (Figure S1a, Supporting Information). Additionally, the unique stacked structure with inserted Au electrode enables VO_2_ NPs to form heterojunctions with graphene and establish additional conductive pathways between source and drain electrodes simultaneously. VO_2_ NPs generate abundant photogenerated carriers due to their remarkable light‐trapping capabilities, which are subsequently separated at the VO_2_/Au barrier to generate significant spontaneous photocurrent (refer to **Figures** **3**a; S1b, Supporting Information). Experimental observations evidenced that the photoresponse of the WE‐ORNT originated from the photovoltaic effect.

Based on the above mechanism, the spectral response of the WE‐ORNT was tested under uniform power density illumination at UV–NIR wavelengths (365–940 nm). As shown in Figure 3b, the device demonstrated a “V‐shaped” wavelength‐dependent photoresponse, with optimal performance achieved at 940 nm. The trend of light response at different wavelengths aligns with the characteristic absorption spectrum of VO_2_ NPs (Figure 2h, black line), indicating their dominant role as the primary photosensitive layer through efficient generation of electron‐hole pairs under illumination. A slight discrepancy between the photocurrent response and absorption spectrum at 590 nm can be attributed to laser‐induced thermal effects. Localized heating induced partial lattice phase transitions in VO_2_, consequently modifying its optical absorption characteristics.^[^ ^50^ ^]^ This interpretation is further supported by the temperature‐dependent absorption spectra (Figure 2h, red line). Figure 3c,d systematically shows the light‐intensity‐dependent photoresponse of WE‐ORNT, quantifying two key performance metrics: photoresponsivity (*R*) and detectivity (D*). These parameters could be evaluated using the following equations:

(1) $$I_{ph}=\left|I_{light}-I_{dark}\right|$$

(2) $$R=\frac{I_{ph}}{P}$$

(3) $$D^{*}=\frac{R\cdot A^{1/2}}{\sqrt{2\cdot e\cdot I_{dark}}}$$

where *I*_ph_ is the net photocurrent of the WE‐ORNT under illumination, defined as the difference between light current (*I*_light_) and dark current (*I*_dark_). *A* represents the photosensitive area (6 × 10^−5^ cm^2^) of the conductive channel. *P* denotes the optical power, obtained through the multiplication of the photosensitive area and the optical power density. *e* is the elementary charge. All photodetection metrics (*R*, D*) of wavelengths from 365 to 940 nm exhibited a reversed trend with illumination power. With increasing incident optical power at 365 nm, the *R* of the WE‐ORNT gradually decreased from 1.83 to 0.95 A W^−1^, while the D* reduced from 1.25 × 10^9^ to 7.95 × 10^8^ Jones. High‐power‐induced performance degradation is predominantly attributed to enhanced carrier recombination by defects.^[^ ^51^, ^52^ ^]^ The WE‐ORNT achieved optimal performance of 3.46 A W^−1^ and 2.50×10^9^ Jones at 940 nm with a power density of 0.71 mW cm^−2^. These parameters demonstrate a significant advantage over most reported self‐powered photodetectors, as shown in Figure S3 (Supporting Information). The photoresponse time is another pivotal metric for optoelectronic switching. Generally, the rise time (*τ*_rise_) and decay time (*τ*_decay_) are defined as the time interval between the 10% and 90% levels of the maximum photocurrent. Compared to conventional transistors, the WE‐ORNT exhibits millisecond‐scale response times across 365, 590, and 940 nm operational wavelengths, shown in Figure 3e–g. In response to 365 nm illumination, the WE‐ORNT demonstrated ultrafast switching kinetics, turning on in 22.3 ms and off in 34.2 ms. Under 590 and 940 nm illumination, the device exhibited *τ*_rise_ of 26.7 and 38.5 ms, and *τ*_decay_ of 35.6 and 37.1 ms, respectively. The response time exhibits pronounced wavelength dependence, indicating that the photoexcited carriers induced by long‐wavelength photons have a lower probability to overcome the Schottky barrier compared to their shorter‐wavelength counterparts. Therefore, the carrier transit time is significantly prolonged, leading to reduced photoresponse speed.^[^ ^53^ ^]^

Repeatable and stable performance is essential for optoelectronic switching devices. The endurance of the WE‐ORNT through 150 photoresponse cycles under 940 nm laser illumination at *V*_ds_ = 0 V is displayed in Figure S4 (Supporting Information). The photocurrent exhibited uniformity without observable degradation, signifying an exceptional stability and repeatability. In contrast, the photocurrent of the pure VO_2_ NPs device shows large fluctuations across trials, suggesting unreliable dynamic behavior (Figure S5, Supporting Information). These phenomena are attributable to discontinuous conductive pathways between source‐drain electrodes, caused by discrete VO_2_ nanoparticles. Conversely, intact monolayer graphene provides continuous conductive channels to the photosensitive VO_2_ layer, ensuring stable photoresponse performance. Furthermore, the optoelectronic behavior of NE‐ORNT without bias was investigated (Figure S6, Supporting Information). Under 940 nm infrared illumination, the NE‐ORNT exhibited rapid optoelectronic switching characteristics similar to those of WE‐ORNTs, indicating its self‐powered photodetection capability. In conclusion, the above experimental results demonstrate the promising potential of the ORNT for energy‐efficient photodetection.

### 2.3 Artificial Optoelectronic Synapse Behaviors of the ORNT

**Figure 4:**
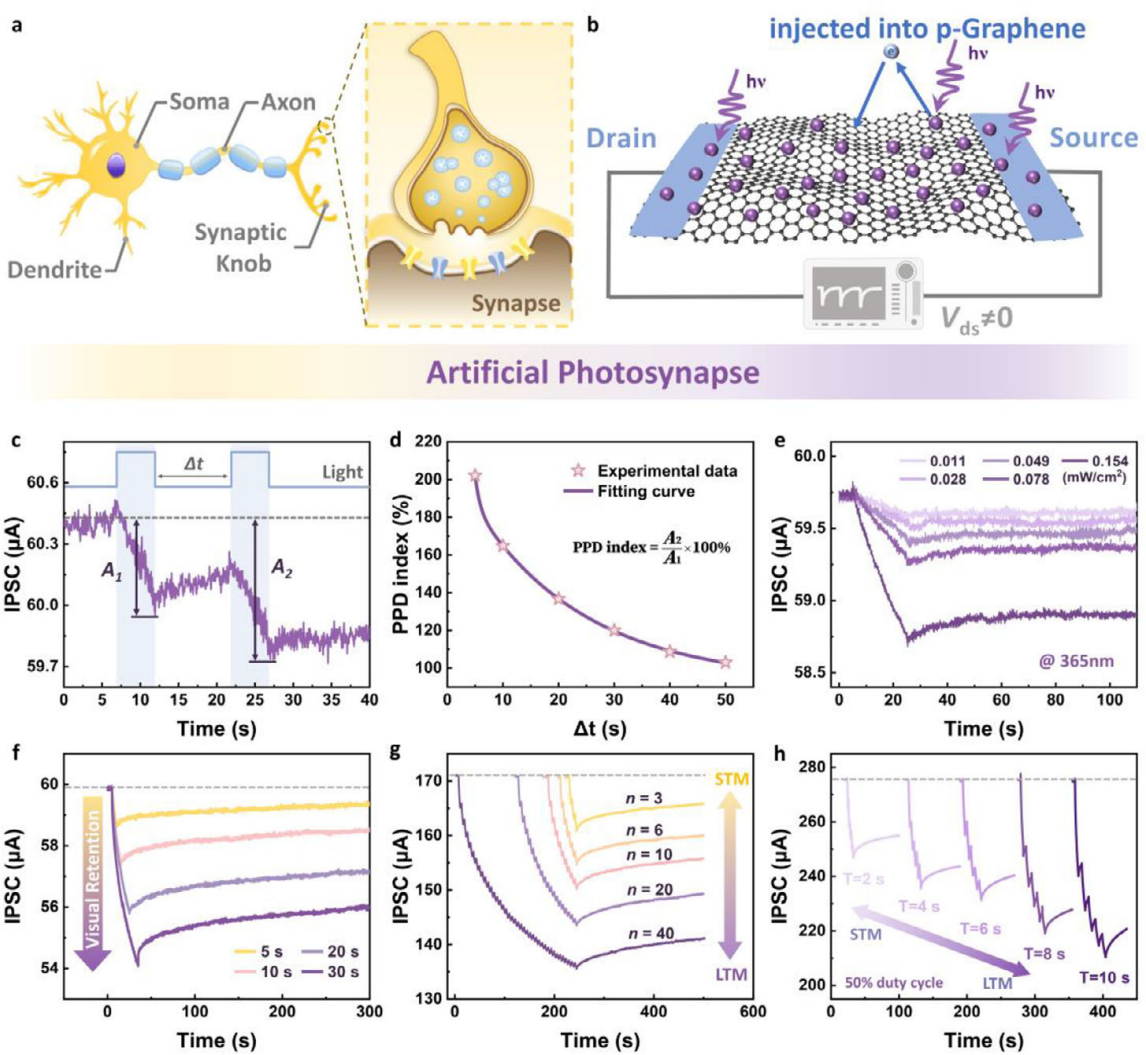
Photo‐tunable synaptic plasticity in WE‐ORNT. a) Corresponding biological synaptic transmission process. The electron transfer process at the heterojunction interface mimics neurotransmitter release in biosynapses. b) The mechanism of WE‐ORNT with photonic synapse functions. c) IPSC triggered by a pair of 365 nm light (1.17 mW cm^−2^) pulses. d) PPD index (star symbols) as a function of interval time, which is fitted by a double exponential function (purple solid line). e) Weak‐light‐triggered short‐term synaptic plasticity. f) SDDP‐mediated visual memory simulation. g) Spike‐number‐dependent plasticity (SNDP) in WE‐ORNT induced by a series of optical pulse stimuli. h) The IPSC generated by a sequence of pulses with different repetition periods (T, duty cycle fixed at 50%). The synaptic performances shown in (f–h) were obtained under irradiation at 365 nm and 15.75 mW cm^−2^.

Synapses serve as the fundamental signal transmission and processing node in the vision neural network. Upon arrival of neural spike trains, postsynaptic potential changes through the release and reception of neurotransmitters. Adaptive potential changes in response to neural activities represent the dynamic adjustment of the functional connection strength between neurons. Such a phenomenon, termed synaptic plasticity, underlies learning and memory. A conceptual illustration is provided in **Figure** **4**a.

In this section, 365 nm UV laser pulses provided presynaptic input signals, while the postsynaptic current (PSC) was simulated by the source‐drain induced current (*I*_ds_). Figure S7 (Supporting Information) illustrates the dynamic characteristics of the WE‐ORNT under an applied bias voltage of 10 mV, where a significantly reduced current is observed under 365 nm UV illumination. Upon light removal, the *I*_ds_ gradually recovered to resting state. This inhibitory synaptic transmission behavior is well‐suited for edge contrast enhancement and background suppression in the visual nervous system. The underlying mechanism arises from the fact that UV (365 nm) irradiation induces ground‐state electron excitation in the photosensitive VO_2_ layer. The generated electron‐hole pairs are subsequently separated by the potential barrier at the graphene/VO_2_ interface. The liberated electrons are then injected into the graphene channel (Figure 3a), where they recombine with rapidly drifting majority holes, leading to a decreased current. Simultaneously, the trapped holes in VO_2_ function as gate‐controlling elements, further modulating the charge carrier concentration through capacitive coupling (as shown in Figure 4b). Under the bias voltage control, the photogating effect enabled the WE‐ORNT to exhibit the key characteristic of synaptic plasticity.

With the underlying mechanism elucidated, the fundamental synaptic functions were systematically investigated in the WE‐ORNT. Figure 4c shows an optically modulated paired‐pulse depression (PPD) process, where a subsequent light stimulus following initial photoexcitation induces a cumulative inhibitory effect on the PSC. This effect is quantified by the PPD index, which is defined as *A*_2_/*A*_1_×100%, where *A*_1_ and *A*_2_ represent the peak intensities of the inhibitory PSC (IPSC) triggered by the first and second optical pulses, respectively. The PPD index, which relies on the pulse interval time (Δ*t*), was fitted using the double exponential function:

(4) $$\mathrm{PPD}/\mathrm{PPF}\mathrm{index}=C_{0}+C_{1}\exp\left(-\frac{\Delta t}{\tau_{1}}\right)+C_{2}\exp\left(-\frac{\Delta t}{\tau_{2}}\right)$$

where *C*_1_ and *C*_2_ are the facilitation constants, *τ*_1_ and *τ*_2_ are the characteristic relaxation times of the two spikes, respectively, and *C*_0_ is the degree of the initial facilitation. As Δ*t* increased from 5 to 50 s, the extracted PPD index decreased from 202.1% to 103.0%, demonstrating a strong interval‐dependent inhibitory plasticity (Figure 4d).

Furthermore, we investigated the weak‐photoexcitation (0.011–0.078 mW cm^−2^, 365 nm) short‐term plasticity. Figure 4e reveals progressively enhanced memory behavior in the light‐intensity‐dependent response of the WE‐ORNT, attributed to the improved photogenerated carrier generation efficiency. The WE‐ORNT maintained a detectable postsynaptic photoresponse even at the minimum illumination intensity as low as 0.011 mW cm^−2^, demonstrating its potential for constructing ultrasensitive and low‐power artificial photonic synapses. Learning and memory efficacy exhibit a positive correlation with repeated rehearsal, like biological systems. The conversion of initial STM into LTM occurs through persistent modulation of artificial synaptic weights by repetitive stimuli. Figure 4f presents the spike‐duration‐dependent plasticity (SDDP) characteristics of the WE‐ORNT exposed to 365 nm light. As the stimulation time increased from 5 to 30 s, the amplitude of PSC suppression rose from ≈1.3 to ≈5.8 µA. Prolonged trapping of massive carriers led to an extended relaxation phase, resulting in elongated optical effect retention that facilitates long‐term visual maintenance. Figure 4g,h investigates the synaptic plasticity dependent on the number and repetition period of spike sequences. Both numerous and prolonged repetitive stimulations drove the WE ‐ ORNT through a learning‐forgetting‐relearning process, ultimately enabling long‐term memory consolidation. In addition, synaptic behaviors under optical excitation at various wavelengths were further characterized, as shown in Figure S8 (Supporting Information). Under applied bias voltage, the WE‐ORNT exhibited photocurrent response and relaxation dynamics across 405–940 nm, enabling the development of bio‐inspired artificial synapses with multiband information fusion capabilities.

**Figure 5:**
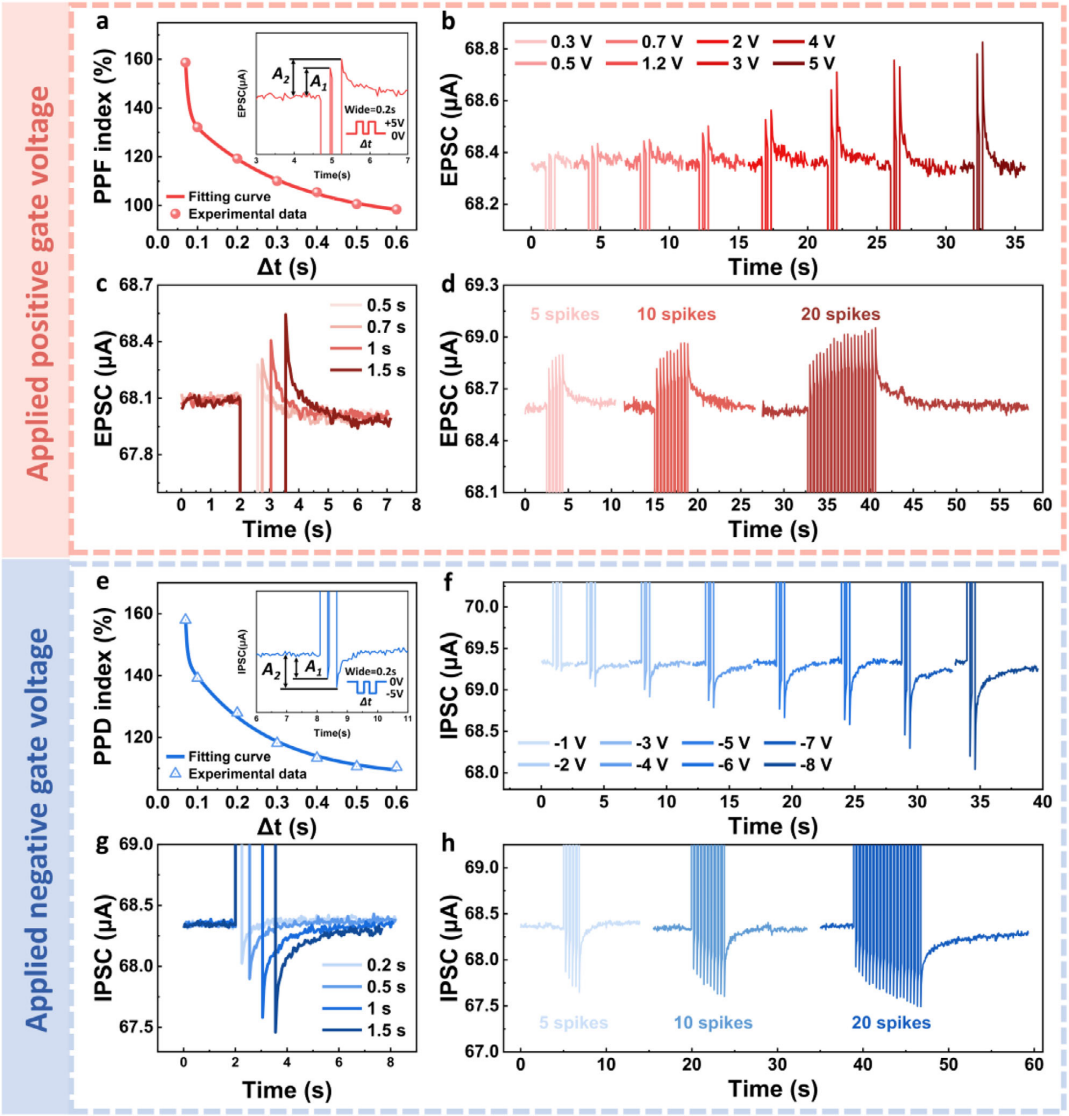
Electronic synapse behaviors of WE‐ORNT. a,e) PPF (spherical symbols) and PPD (triangle symbols) index as a function of interval time, which are fitted with the double exponential functions (red and blue solid line). Insets are the EPSC and IPSC under paired‐pulse stimulation, respectively. b) spike‐voltage‐dependent plasticity (SVDP) under gate pulse stimulation with amplitudes ranging from 0.3 to 5 V and a fixed 0.2 s pulse width and interval. c) SDDP under positive gate electric stimulation with pulse width from 0.5 to 1.5 s and a 5 V fixed pulse amplitude. d) SNDP under positive gate electric stimulation with fixed 3 V amplitude, 0.2 s pulse width, and 0.2 s interval. f) SVDP under gate pulse stimulation with amplitudes ranging from −1 to −8 V and a fixed 0.2 s pulse width and interval. g) SDDP under negative gate pulse stimulation with a pulse width from 0.2 to 1.5 s and a −5 V fixed pulse amplitude. h) SNDP under negative gate electric stimulation with fixed −5 V amplitude, 0.2 s pulse width, and 0.2 s interval.

In addition to photonic synapses, gate‐tunable bipolar artificial electrical synapses also represent a critical approach for emulating multimodal signals of biological neurotransmission. Presynaptic neuronal spikes were converted into sequential electrical potentials applied to the gate electrode. The insets in Figure 5a,e illustrate the device's excitatory PSC (EPSC) and IPSC responses under gate voltages of 5 and −5 V, respectively, with 0.2 s pulse width and 0.1 s interval. Under gate voltage, the PSC exhibited abrupt transitions during gate‐voltage spikes due to field effects, followed by rapid retraction with overshoot upon pulse removal. This overshoot is likely attributed to the rapid release of charges trapped in the dielectric layer or interface, which generates an additional electric field opposite to the original gate electric field, temporarily enhancing the channel conductivity. Thereafter, the relaxation of deep‐level traps and carrier recombination leads to the slow recovery of the current. Similarly, the paired‐pulse facilitation (PPF) and PPD index for the electronic synapse were quantified through calculation and fitting (refer to Equation 4), with the results shown in Figure 5a,e. The PPF and PPD indices reached maximum values of 158.7% and 158.0% at pulse intervals of 0.07 s, respectively. Based on the fitting results, *τ*_1_ and *τ*_2_ were determined to be 7.3 and 252.5 ms for PPF, and 4.6 and 197.3 ms for PPD, respectively. These values are consistent with the biological synaptic time constants, which typically range from milliseconds to seconds. Similar to photonic synapses, electronic synapses exhibit typical bipolar (excitatory and inhibitory) synaptic plasticity through the modulation of amplitude, duration, and pulse number for the gate voltage (Figure 5b–d and f–h). Repeated input of salient information induces extended forgetting curves, demonstrating enhanced memory retention. It is worth noting that negative gate pulses of equal magnitude resulted in enhanced field‐effect channel currents and overshoot currents compared to positive pulses (Figure S9, Supporting Information). This asymmetric response stems from the inherent p‐type doping of graphene within the channel. Moreover, we implemented light‐induced inhibitory (programming) and voltage‐controlled potentiation (erasing) of the device through a series of optoelectronic pulse stimulation. In Figure S10 (Supporting Information), as 20 UV laser pulses (365 nm, 0.38 mW cm^−2^) were continuously applied, the conductivity of WE‐ORNT gradually decreased to ≈3.2 mS. Erasing those relaxation effects required 17 consecutive (3 V, pulse width = 0.2 s, interval = 0.1 s) electrical pulses to restore the initial state. These behaviors of the WE‐ORNT are expected to be more relevant to spiking neural networks (SNN) computing.

### 2.4 Photonic Storage Characteristics of the ORNT

**Figure 6:**
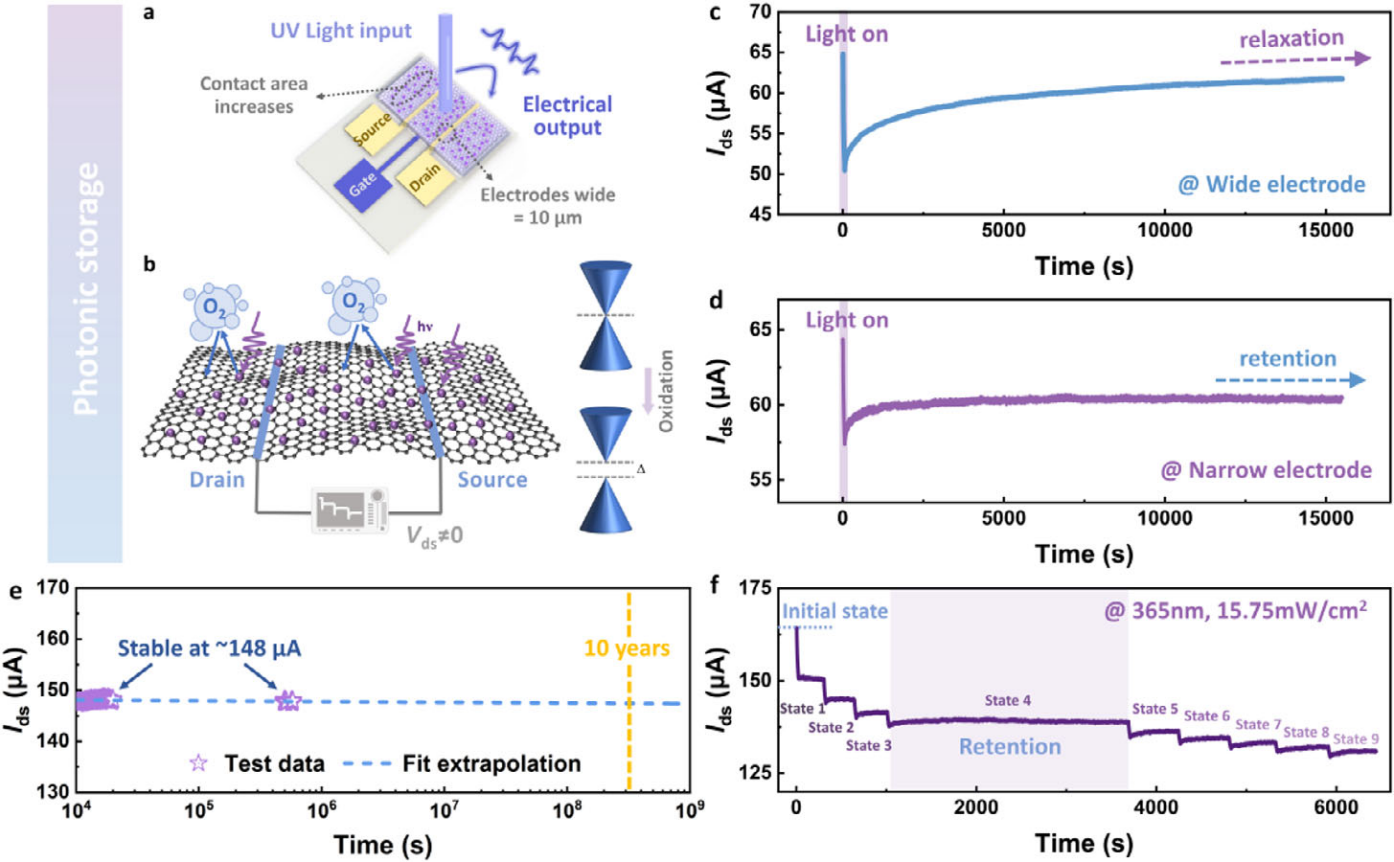
The nonvolatile photonic storage properties of the NE‐ORNT. a) Schematic illustration of the narrow‐electrode optimized device. b) Proposed storage mechanism and comparative band diagrams of graphene before and after oxidation. c,d) The *I*_ds_ characteristics of ORNTs with varying electrode widths under identical illumination conditions (365 nm, 15.75 mW cm^−2^). e) Retention and endurance performance of narrow electrode‐based storage ORNT. f) Discrete conductance states in NE‐ORNT achieved through multistage photomodulation.

Storage represents the final stage in the visual processing hierarchy. In electronic implementations, materials such as oxides,^[^ ^54^, ^55^, ^56^ ^]^ sulfur compounds,^[^ ^57^, ^58^ ^]^ and organic molecules^[^ ^59^ ^]^ undergo local structural or electronic state changes when irradiated by light pulses. These changes include oxygen vacancy defects, phase transitions, charge trapping and release, and so on. As a result, discrete resistance values appear, corresponding to different storage states. Inspired by the aforementioned mechanism, the source‐drain electrode dimensions of the ORNT were optimized to achieve the storage effect. **Figure** **6**a presents the schematic diagram of the NE‐ORNT. The *I*_ds_ characteristics of devices were tracked with varying electrode widths under identical illumination conditions (365 nm, 15.75 mW cm^−2^), as shown in Figure 6c,d. Under illumination, the photocurrent of WE‐ORNT rapidly decreased to ≈50.4 µA followed by slow relaxation to the resting potential, exhibiting synaptic‐like dynamics. In contrast, NE‐ORNT maintained a stable post‐illumination current (≈60.3 µA) with minimal relaxation, exhibiting storage characteristics. This is caused by the oxidation of graphene by oxygen released during the photoinduced phase transition of VO_2_. Further characterization was conducted to validate the involved physical mechanisms. Figure S11 (Supporting Information) presents split‐peak fitting results of O 1s X‐ray photoelectron spectroscopy in VO_2_ under identical illumination conditions (365 nm, 15.75 mW cm^−2^) with varying exposure durations (0, 5, 10, and 20 min). The large specific surface area of VO_2_ NPs promoted stronger light‐matter interactions, resulting in a 4.55% rise of vacancy‐oxygen content after only 5 min irradiation. With prolonged irradiation to 20 min, the vacancy‐oxygen content increased to 31.37% (a rise of 13.94%), as quantified by XPS peak area integration. This phenomenon demonstrates that 365 nm UV irradiation induces oxygen release in VO_2_ crystal lattice, generating oxygen vacancies, which is consistent with those reported in prior studies.^[^ ^54^, ^60^ ^]^ In the optimized NE‐ORNT, the graphene/VO_2_ interfacial contact area increases by approximately seven times (Figure 2d), significantly enhancing the probability of carbon‐oxygen bonding between the graphene surface and oxygen. This interfacial oxidation disrupts the π‐electron conjugation and pristine sp^2^ carbon network, elevating graphene's resistance through impaired charge transport,^[^ ^61^, ^62^ ^]^ which ultimately preserves the effect of UV illumination as reflected in the current. Figure 6b illustrates the proposed storage mechanism, along with comparative band diagrams of graphene before and after oxidation. Concurrently, the appearance of oxygen vacancies leads to electronic and structural phase transitions in the VO_2_ lattice.^[^ ^63^ ^]^

To validate the nonvolatility and reliability of the NE‐ORNT, retention and endurance tests were recorded and visually presented in Figures 6e and S12 (Supporting Information). After 20 s of laser stimulation at 365 nm, the NE‐ORNT maintained a stable current of ≈147.9 µA for over 10^5^ s without observable degradation. Linear fitting and extrapolation of the decay kinetics predict data retention exceeding 10 years. Based on this, the NE‐ORNT photonic storage device could be further modulated. Figure 6f displays the *I*_ds_ behavior under alternating cycles of 365 nm UV irradiation at 15.75 mW cm^−2^ and dark conditions. When the NE‐ORNT was illuminated, the *I*_ds_ state changed rapidly; upon light removal, the *I*_ds_ stabilized to a plateau, corresponding to the encoding of a persistent state. Nine reliable multilevel storage states were achieved. State 4′s dark current behavior was tracked over time, confirming nonvolatile retention across all programmed states following multistage photomodulation. Collectively, NE‐ORNT‐based photonic storage demonstrates promising potential for processing‐in‐memory applications, highlighting its viability for next‐generation computing architectures.

### 2.5 ORNT‐Based Optical Communication and Processing‐in‐Memory System

**Figure 7:**
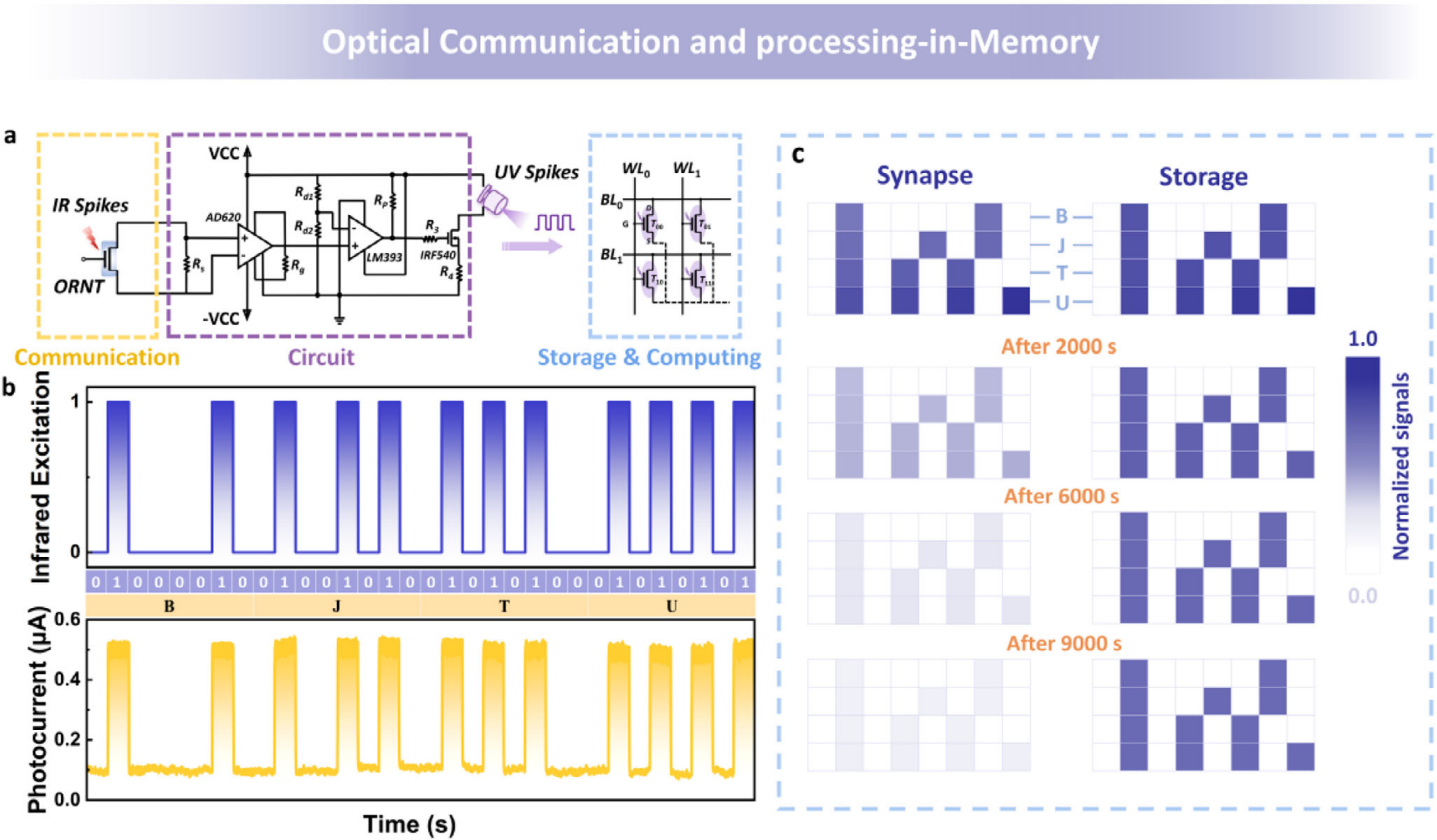
Integrated optical communication and processing‐in‐memory system based on the ORNT. a) Conceptual circuit diagram of the system. b) The output photocurrent signal of the ASCII code “BJTU” by ORNT. c) Time‐sequential UV (365 nm, 15.75 mW cm^−2^) writing of the four ASCII codes for “B,” “J,” “T,” and “U.” The shade of color changes represents normalized Δ*I* of ORNTs after 2000, 6000, and 9000 s.

Consequently, an integrated optical communication and processing‐in‐memory system was proposed based on the ORNT's novel reconfigurable properties and superior neuromorphic behavior. The conceptual circuit diagram of the system is presented in **Figure** **7**a. In the optical communication module, a sequence of 940 nm optical pulses (2.83 mW cm^−2^) modulated with the ASCII encoding of “BJTU” was applied to the ORNT without external bias voltage, as demonstrated in Figure 7b. Upon optical stimulation, the ORNT promptly generated a photocurrent that precisely matched the ASCII encoding of “BJTU,” which could then be decoded for subsequent data processing. When the photocurrent generated by the ORNT under infrared excitation exceeded the preset threshold of the circuit, UV light pulses were triggered. These UV light pulses were used to sequentially program the WE and NE devices of the ORNT, which exhibited synaptic and memory properties. As shown in Figure 7c, each row corresponds to the time‐sequential UV writing of the four ASCII codes for “B,” “J,” “T,” and “U,” respectively. Normalized channel current variations (Δ*I*, scaled between 0 and 1) were represented by varying color intensities for enhanced visual comparison. During the initial input phase, the ORNTs with both synaptic and storage properties exhibited similar decreases in current responses. After 2000 s of encoding completion, the synaptic ORNT demonstrated significant chromatic degradation compared to the storage device. After 6000 s, the UV‐encoded synapse relaxed to near its initial state, while the storage device maintained a locked Δ*I*. The aforementioned process demonstrates that the ORNT displays exceptional reconfigurable neuromorphic characteristics across different operational conditions, offering efficient technological support for integrated optical communication and processing‐in‐memory applications.

## 3 Conclusion

In summary, a novel optoelectronic reconfigurable unit based on an Au‐electrode‐inserted graphene/VO_2_ heterostructure was demonstrated, which emulates critical perception, computation, and nonvolatile storage functionalities of biological visual systems through mode switching. The ORNT exhibits broad‐spectrum photodetection capability ranging from UV to NIR (365–940 nm). Benefiting from the light‐absorption in the VO_2_ layer and photovoltaic effect, a remarkable photoresponsivity of 3.46 A W^−1^, a fast photoresponse speed of *τ*_rise_/*τ*_decay_ with 22.3/34.2 ms, and a high detectivity of 2.50×10^9^ Jones were achieved in WE‐ORNT at zero bias. The diverse synaptic plasticity (PPF/PPD/SDDP/SNDP/SVDP) in the WE‐ORNT arises from the photogating‐dominated effect coupled with the field‐effect modulation under applied bias voltages. Electrode optimization combined with photoinduced phase transition of VO_2_ enabled NE‐ORNTs to achieve both high robustness in data retention (theoretical endurance >10 years) and multilevel optical storage capacity. Furthermore, we designed an integrated system that synergistically combines infrared optical communication with ultraviolet processing‐in‐memory operations. This design not only addresses the critical need for adaptive hardware resource allocation and extension, but also pioneers a new pathway toward light‐speed perception‐computation‐storage convergence, emulating biological vision systems.

## 4 Experimental Section

### Fabrication of Reconfigurable Graphene/VO_2_ NPs Devices

First, the 4‐inch silicon substrate was sonicated with H_2_SO_4_: H_2_O_2_ (Volume ratio 4:1) mixed solution for 30 min to clean the surface and then dried with high‐purity nitrogen. Next, the buried gate electrode Cr/Au (10/50 nm) was fabricated by lithography and magnetron sputtering. CVD‐grown monolayer graphene was transferred onto a 50‐nm thick SiO_2_ dielectric layer prepared by ICPECVD and soaked in acetone to remove the PMMA. Areas outside of the pattern were etched away by ICP‐F and plasma. Subsequently, the source and drain electrodes were defined onto the monolayer graphene by standard lithography, followed by Electron‐beam evaporation of Cr/Au (10/50 nm) films and a 30‐min lift‐off process in acetone. Commercial VO_2_ nanoparticles were ground, alcohol‐dispersed (25 mg mL^−1^), and centrifuged (8000 rpm, 4 min) to obtain a suspension, which was drop‐cast onto the device surface.

### Optoelectronic Reconfiguration Measurements

Electrical characteristics (*V*_ds_/*I*_ds_/*V*_gs_) of reconfigurable devices were monitored on the prober using a semiconductor parameter analyzer (B1500A, Keysight). Light pulses were generated by a laser (CEL‐LEDS35, CEAULIGHT). The illumination power was adjusted by the potential attenuator and calibrated by a standard optical power meter (LP10, Sanwa). All the optoelectronic reconfiguration measurements were conducted in a dark ambient environment with a temperature of 25 °C.

### Characterizations

The dimensions and surface morphology of devices were obtained by the scanning electron microscope (SU8220, HITACHI). Transmission electron microscopy (2100F, JEOL) magnified the details of VO_2_ nanoparticles, which have significant implications for photoresponse. X‐ray photoelectron spectroscopy (Thermo Scientific K‐Alpha) was used to identify the oxygen vacancy states of VO_2_ nanoparticles before and after UV illumination. The obtained binding energy was calibrated with a C 1s peak at 248.8 eV. The crystal structure of the drop‐casted VO_2_ NPs was characterized by X‐ray diffraction (D8 Advance, Bruker). A UV–vis–NIR spectrophotometer (PE Lambda950) was used to measure the optical absorbance of VO_2_ NPs.

## Conflict of Interest

The authors declare no conflict of interest.

## Supporting information



Supporting Information

## Data Availability

The data that support the findings of this study are available from the corresponding author upon reasonable request.
