# Supplementary material for: Novel Optoelectronic Reconfigurable Transistors Based on Graphene/VO2 Heterojunction for Efficient Neuromorphic Perception, Computation, and Storage
Source: Adv Sci (Weinh). 2025 Sep 26;12(46):e13429. doi: 10.1002/advs.202513429 (PMC12697805; doi:10.1002/advs.202513429)
Supplement: Supplementary file 1 — Supporting Information [file ADVS-12-e13429-s001.docx]

**Supporting Information for**

**Novel Optoelectronic Reconfigurable Transistors based on Graphene/VO_2_ Heterojunction for Efficient Neuromorphic Perception, Computation and Storage**

Danke Chen^1^, Yuning Li^1^, Xiaoqiu Tang^1^, Jingye Sun^1^, Xuan Yao^1^, Peizhi Yu^2^, Xue Li^1^, Qing You^1^, Hanyu Wang^1^, He Tian^*,3^, Tao Deng^*,1^

^1^School of Electronic and Information Engineering, Beijing Jiaotong University, Beijing, 100044, People’s Republic of China

^2^Department of Precision Instrument, Tsinghua University, Beijing 100049, People’s Republic of China

^3^School of Integrated Circuits and Beijing National Research Center for Information Science and Technology (BNRist), Tsinghua University, Beijing 100049, People’s Republic of China

^*^ e-mail: He Tian, tianhe88@tsinghua.edu.cn; Tao Deng, dengtao@bjtu.edu.cn

This PDF file includes：

Figure S1 to S12 (Page 1-6)

Table S1 (Page 7)

Reference (Page 8-9)





**Figure S1.** The normalized photocurrent of (a) monolayer graphene and (b) graphene/VO_2_ heterojunction devices under 940 nm infrared light illumination at *V*_ds_ = 0 V.


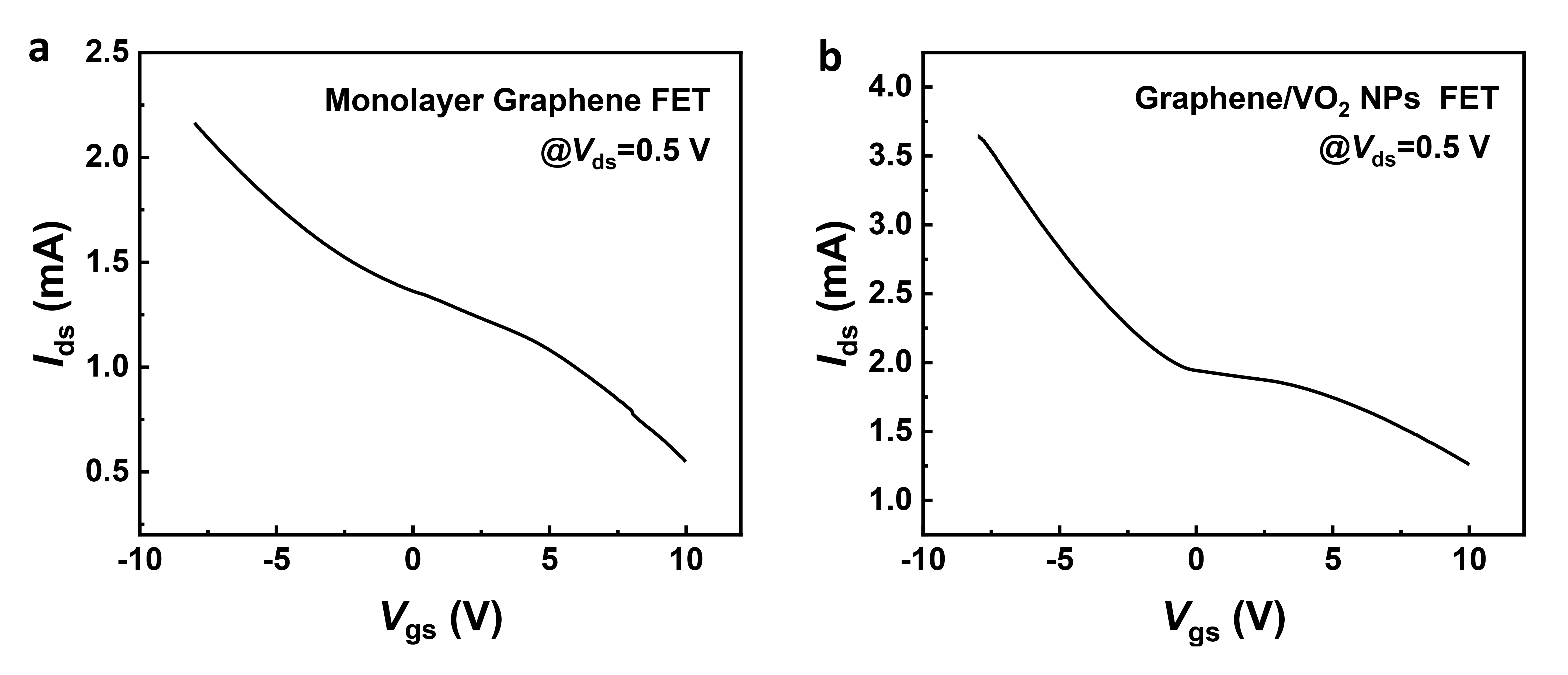


**Figure S2.** The transfer characteristic curves of (a) monolayer graphene and (b) graphene/VO_2_ heterojunction field effect transistors at *V*_ds_ = 0.5 V.

No discernible Dirac point was observed, potentially attributed to p-type doping induced by ions and water molecules introduced during the procedure. The maximum scan gate voltage was limited to 10 V to prevent dielectric breakdown that could occur at higher voltages.


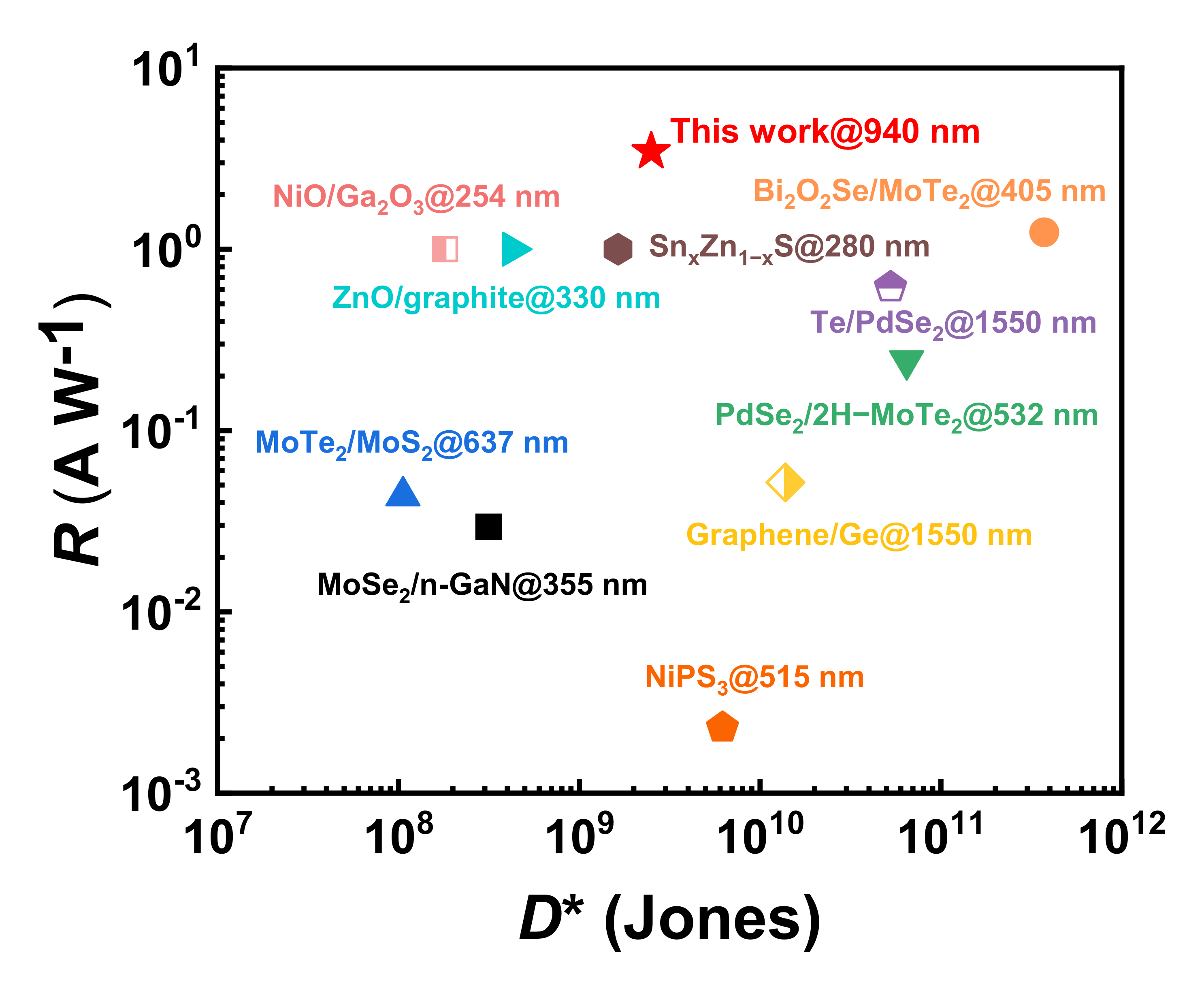


**Figure S3.** Comparison of *R* and *D** of self-powered photodetectors^1-10^.


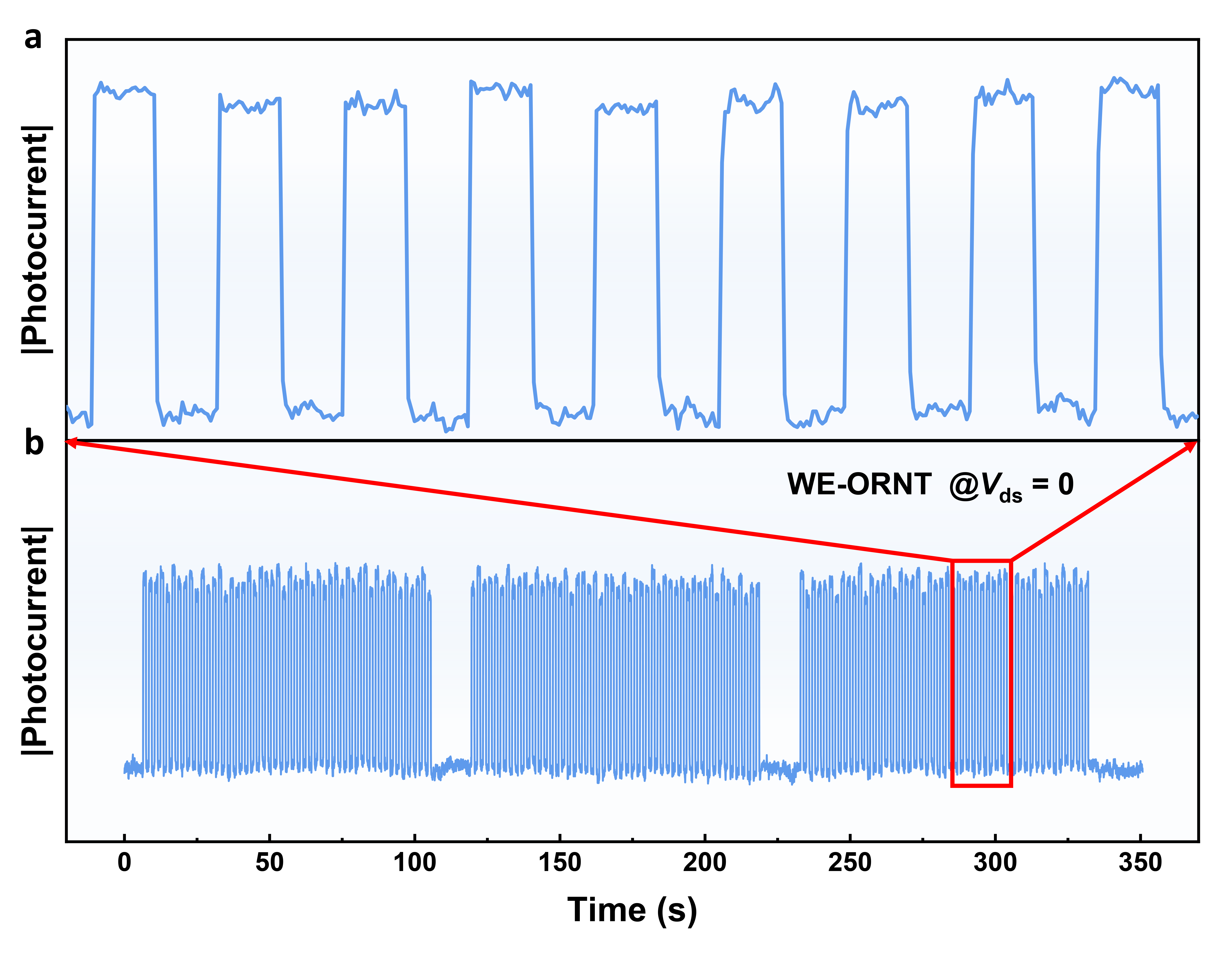


**Figure S4.** (a) The enlarged view of the partial response current in (b). (b) The stability of the WE-ORNT within 150 photoresponse cycles under 940 nm light illumination at *V*_ds_ = 0 V.


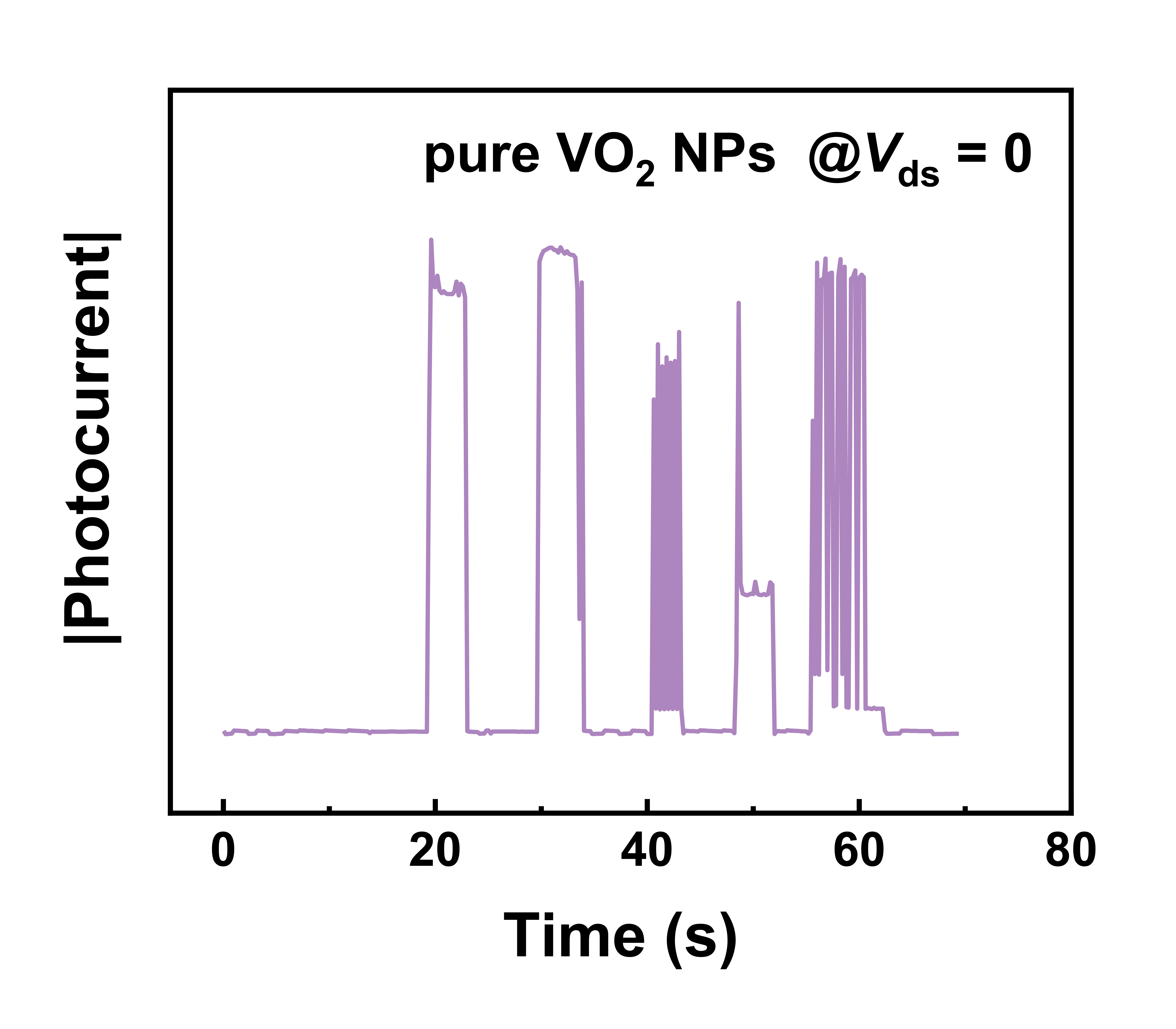


**Figure S5.** The photocurrent for pure VO_2_ nanoparticles device under 940 nm light illumination at *V*_ds_ = 0 V.


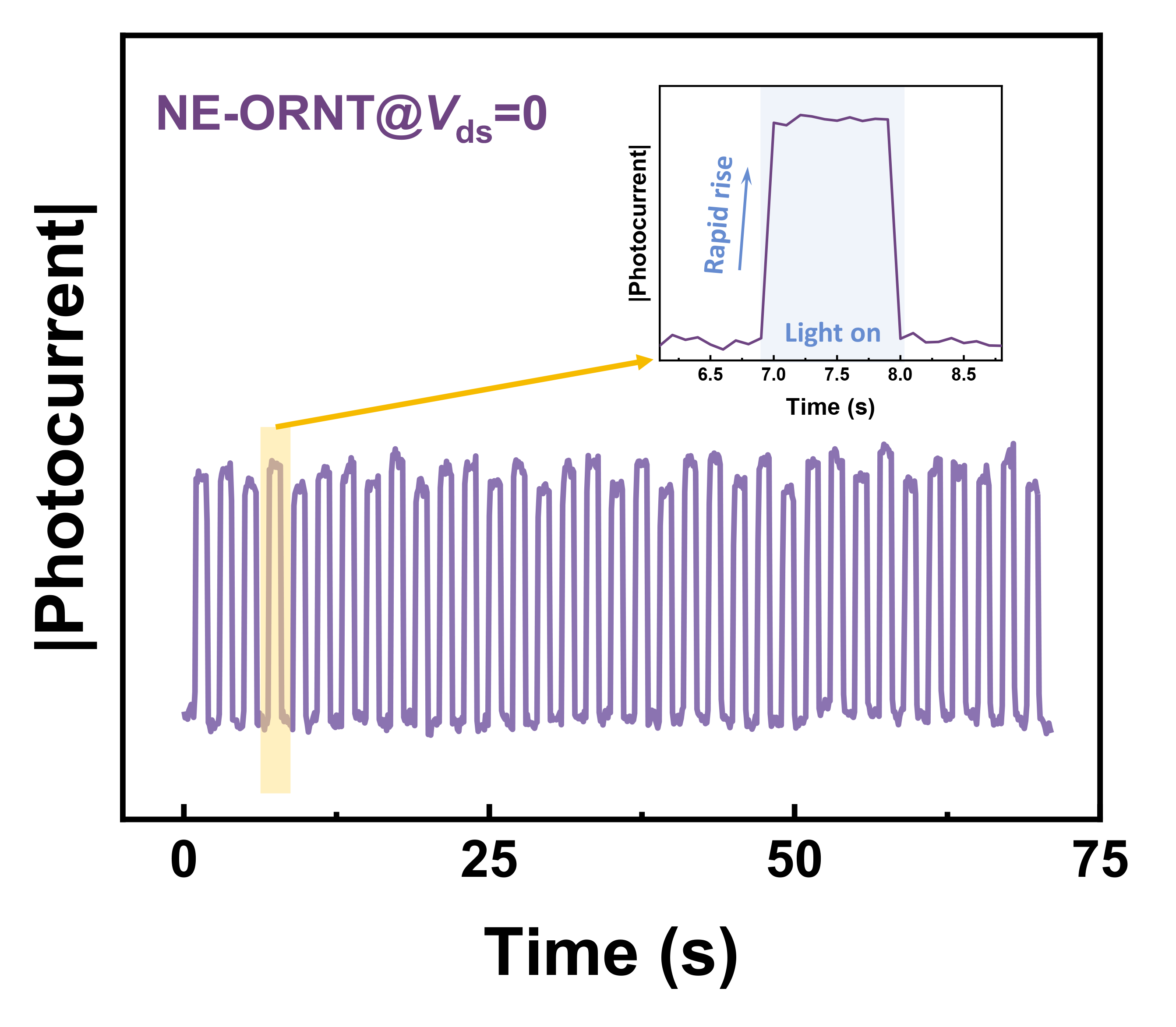


**Figure S6.** The optoelectronic characteristics of the narrow-electrode ORNT (NE-ORNT) under 940 nm light illumination at *V*_ds_ = 0 V.


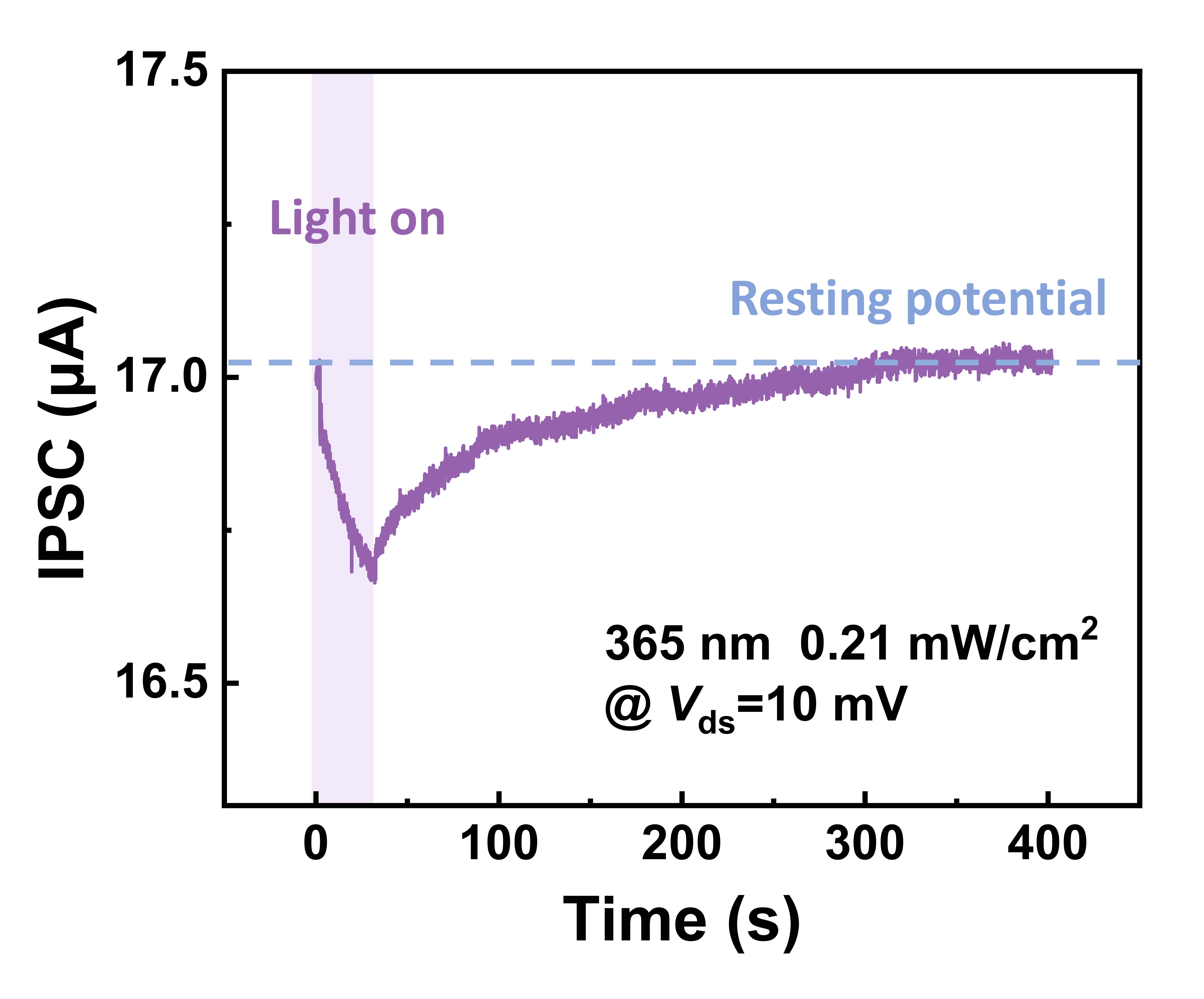


**Figure S7.** Light-induced synaptic inhibition behavior in the WE-ORNT under 10 mV bias voltage.


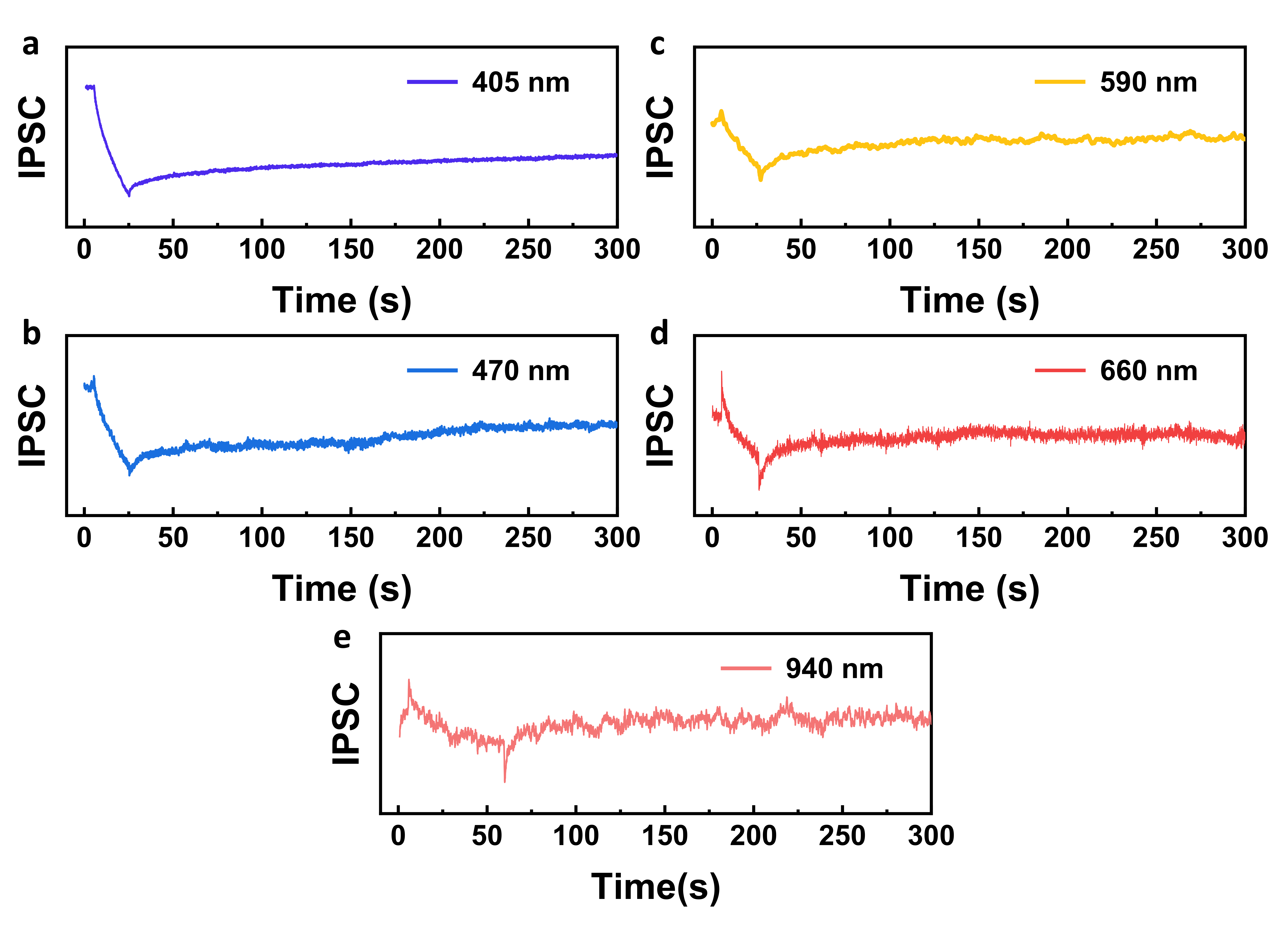


**Figure S8.** (a-e) Synaptic behaviors of WE-ORNTs under 405nm to 940nm laser stimulations.

Previous studies have confirmed that the activation energy of oxygen vacancies in VO_2_ ranges between 3 and 3.5 eV, enabling photon-induced phase transition under higher-energy illumination^11,12^. This phase transition elevates the Fermi level of VO_2_, synergizing with the photogating effect to facilitate electron migration into graphene and subsequent recombination with majority hole carriers in the channel. This process leads to a further reduction in postsynaptic current (PSC). In contrast, under lower-energy illumination (e.g., 405 ~ 940 nm wavelengths), the photogating effect remains the dominant mechanism governing synaptic behavior.


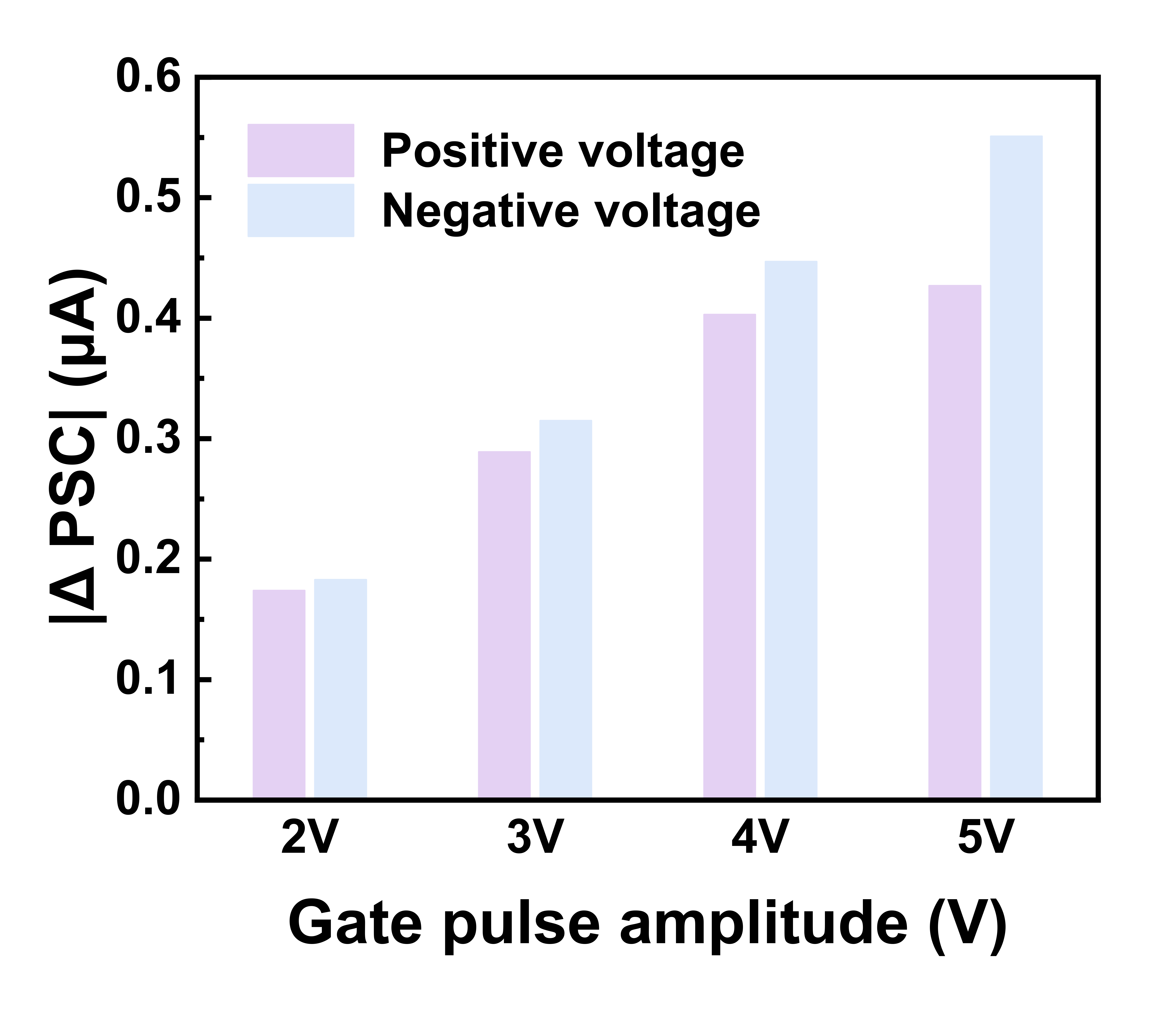


**Figure S9.** The overshoot currents amplitude (ΔPSC) of gate polarity-dependent synaptic plasticity. Facilitation under 2 V~5 V (purple stripes) vs Suppression under −2 V ~ −5 V (blue stripes).


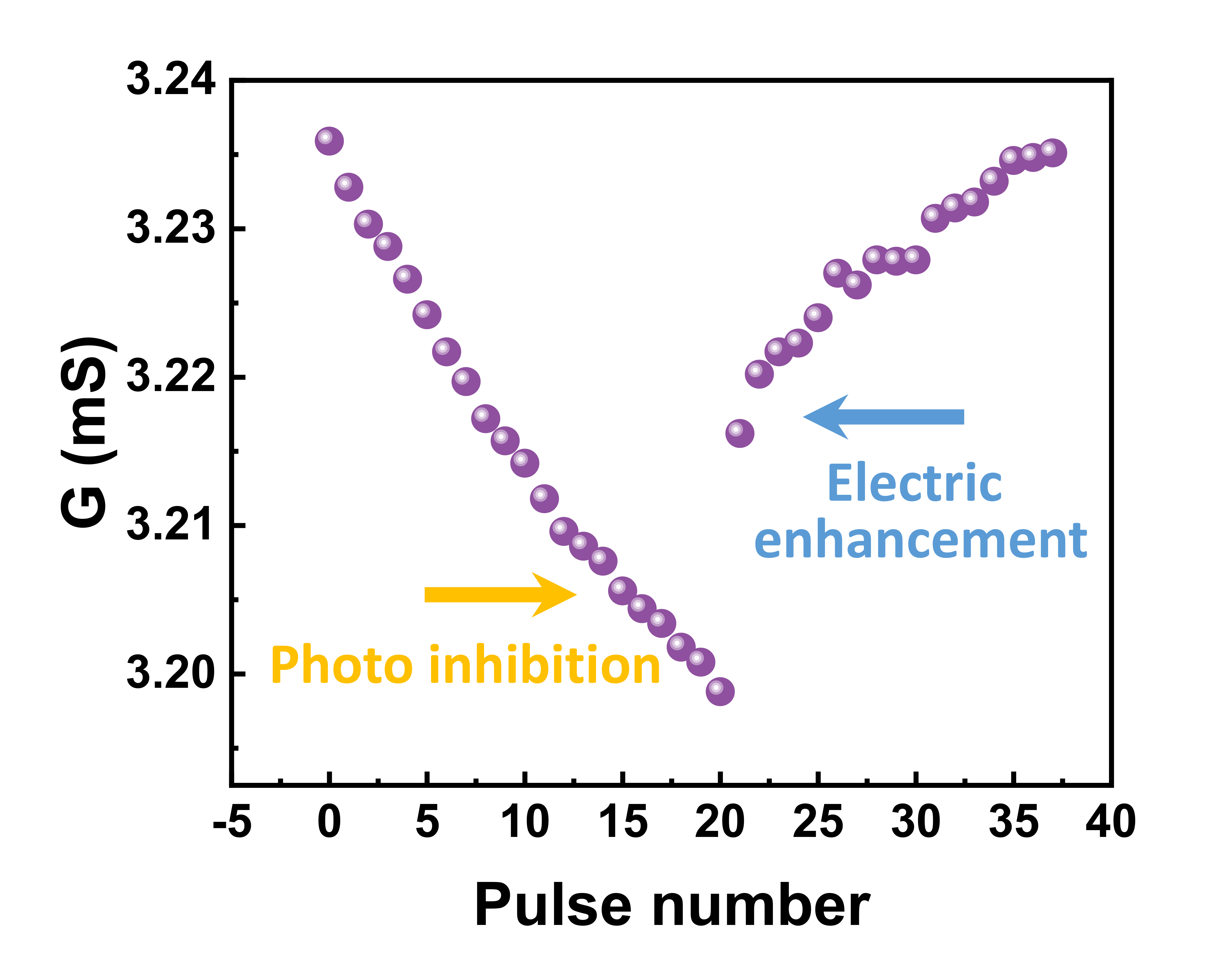


**Figure S10.** Continuous modulations of the inhibitory and potentiation properties of the WE-ORNT by sequential optical and electrical pulse trains.





**Figure S11.** Split-peak fitting analysis of O 1s X-ray photoelectron spectroscopy in VO_2_ under 365 nm illumination (15.75 mW cm^-2^) at (a) 0 min, (b) 5 min, (c) 10 min, (d) 20 min varying exposure durations. O_ads_, O_vac_, and O_latt_ represent adsorbed oxygen, oxygen vacancy, and lattice oxygen, respectively.





**Figure S12.** Retention and endurance characteristics of storage-enabled NE-ORNT under continuous readout (*V*_ds_ = 50 mV).

**Table S1.** Comparison of the reconfigurable neuromorphic device characteristics

| Materials | Structure | Reconfigurable mechanism | | Application | Optoelectronic cooperation (Yes/No) | Bandwidth | Ref. |
| --- | --- | --- | --- | --- | --- | --- | --- |
| ITO/(PVA-Mxene)/Ag | FET | EDL ^a^ effect/ECM ^b^ | synapse/neuron | | N | / | ^13^ |
| α-In_2_Se_3_ | FET | polarization effects | synapse/neuron | | N | / | ^14^ |
| Ag/SiC/n-Si | Two-terminal | ECM (adjusting the current compliance) | synapse/neuron | | N | / | ^15^ |
| Ag/MoS_2_/HfAlO_x_/CNT | Two-terminal (electronic textiles) | ECM (adjusting the current compliance) | synapse/neuron | | N | / | ^16^ |
| Graphene/HIE ^c^ | FET | gate-controlled  electrochemical reactions | synapse/neuron | | N | / | ^17^ |
| MoTe_2_ | FET | EGV-pGD ^d^ | diode/memory/synapse | | Y | white-light | ^18^ |
| h-BN/Graphene/MoS_2_ | FET | photovoltaic effect /ECM/ photogating effect | synapse/neuron/ dendrite | | Y | 532 nm | ^19^ |
| Graphene/h-BN/MoTe_2_//MoS_2_ | FET | Gate control  /photogating effect | sensor/memory/synapse | | Y | 635nm | ^20^ |
| ITO/Al_2_O_3_/HfSe_2_  /Al_2_O_3_/p-Si | Two-terminal | Charge trapping/*V*_FB_ ^e^ shift and memcapacitor | synapse/neuron | | Y | 465~785 nm | ^21^ |
| TiN/Ti/HfO_2_/TiN | Two-terminal | VCM ^f^ | synapse/neuron | | N | / | ^22^ |
| Graphene/VO_2_ | FET | photovoltaic effect/ photogating effect/  photoinduced phase transition | switching/synapse  switching/storage | | Y | 365~940 nm | This work |

^a^ EDL: electric double layer

^b^ ECM: electrochemical metallization

^c^ HIE: hydrogen ion electrolyte

^d^ EGV-pGD: effective-gate-voltage-programmed graded-doping

^e^ *V*_FB_: flat-band voltage

^f^ Valence change mechanism

**Supplementary References:**

1. J. Yu, M. Yu, Z. Wang, et al., “Improved Photoresponse Performance of Self-Powered β-Ga₂O₃/NiO Heterojunction UV Photodetector by Surface Plasmonic Effect of Pt Nanoparticles,” *IEEE Transactions on Electron Devices* 67, no. 8 (2020):3199-3204. doi:10.1109/ted.2020.2999027

2. Z. Dan, B. Yang, Q. Song, et al., “Type-II Bi_2_O_2_Se/MoTe_2_ van der Waals Heterostructure Photodetectors with High Gate-Modulation Photovoltaic Performance,” *ACS Applied Materials & Interfaces* 15, no. 14 (2023):18101-18113. doi:10.1021/acsami.3c01807

3. R. Sinha, N. Roy, T. Mandal, “Growth of Carbon Dot-Decorated ZnO Nanorods on a Graphite-Coated Paper Substrate to Fabricate a Flexible and Self-Powered Schottky Diode for UV Detection,” *ACS Applied Materials & Interfaces* 12, no. 29 (2020):33428-33438. doi:10.1021/acsami.0c10484

4. S. Ebrahimi, B. Yarmand, “Solvothermal growth of aligned Sn_x_Zn_1-x_S thin films for tunable and highly response self-powered UV detectors,” *Journal of Alloys and Compounds* 827, (2020): 154246. doi:10.1016/j.jallcom.2020.154246

5. P. Wang, Z. Li, X. Xia, et al., “Anisotropic Te/PdSe_2_ Van Der Waals Heterojunction for Self‐Powered Broadband and Polarization‐Sensitive Photodetection,” *Small* 20, no. 34 (2024): 2401216. doi:10.1002/smll.202401216

6. M. Che, B. Wang, X. Zhao, et al., “PdSe_2_/2H–MoTe_2_ Heterojunction Self-Powered Photodetector: Broadband Photodetection and Linear/Circular Polarization Capability. *ACS Nano* 18, no 44 (2024):30884-30895. doi:10.1021/acsnano.4c12298

7. Y. Chen, X. Wang, G. Wu, et al., “High‐Performance Photovoltaic Detector Based on MoTe2/MoS2 Van der Waals Heterostructure,” *Small* 14, no. 9 (2018): 1703293. doi:10.1002/smll.201703293

8. L. Zeng, M. Wang, H. Hu, et al., “Monolayer Graphene/Germanium Schottky Junction As High-Performance Self-Driven Infrared Light Photodetector,” *ACS Applied Materials & Interfaces* 5, no. 19 (2013):9362-9366. doi:10.1021/am4026505

9. R. Kumar, B. Singh, V. Aggarwal, et al., “Self-powered broadband ultraviolet photodetector based on MoSe_2_/n-GaN heterojunction,” *Journal of Alloys and Compounds* 1014, (2025): 178813. doi:10.1016/j.jallcom.2025.178813

10. L. Zong, J. Song, S. Wang, et al., “A broadband self-powered photodetector based on NiPS_3_,” *Journal of Materials Chemistry C* 12, no. 2 (2024):593-599. doi:10.1039/d3tc03804a

11. H. Zhang, L. Guo, G. Stone, et al., “Imprinting of Local Metallic States into VO_2_ with Ultraviolet Light,” *Advanced Functional Materials* 26, no. 36 (2016):6612-6618. doi:10.1002/adfm.201601890

12. X. Yu, C. Cheng, J. Liang, et al., “Graphene‐Assisting Nonvolatile Vanadium Dioxide Phase Transition for Neuromorphic Machine Vision,” *Advanced Functional Materials* 34, no. 16 (2024): 2312481. doi:10.1002/adfm.202312481

13. X. Zhang, S. Wu, R. Yu, et al., “Programmable neuronal-synaptic transistors based on 2D MXene for a high-efficiency neuromorphic hardware network,” *Matter* 5, no. 9 (2022):3023-3040. doi:10.1016/j.matt.2022.06.009

14. Y. Zhai, P. Xie, J. Hu, et al., “Reconfigurable 2D-ferroelectric platform for neuromorphic computing,” *Applied Physics Reviews* 10, no. 1 (2023): 011408. doi:10.1063/5.0131838

15. X. Yan, Y. Shao, Z. Fang, et al., “A low-power reconfigurable memristor for artificial neurons and synapses,” *Applied Physics Letters* 122, no. 4 (2023): 042101. doi:10.1063/5.0128200

16. T. Wang, J. Meng, X. Zhou, et al., “Reconfigurable neuromorphic memristor network for ultralow-power smart textile electronics,” *Nature Communications* 13, no. 1 (2022): 7432. doi:10.1038/s41467-022-35160-1

17. C. Yu, S. Li, Z. Pan, et al., “Gate-Controlled Neuromorphic Functional Transition in an Electrochemical Graphene Transistor,” *Nano Letters* 24, no. 5 (2024):1620-1628. doi:10.1021/acs.nanolett.3c04193

18. R. Peng, Y. Wu, B. Wang, et al., “Programmable graded doping for reconfigurable molybdenum ditelluride devices,” *Nature Electronics* 6, no. 11 (2023):852-861. doi:10.1038/s41928-023-01056-1

19. J. Hu, H. Li, Y. Zhang, et al., “Reconfigurable Neuromorphic Computing with 2D Material Heterostructures for Versatile Neural Information Processing,” *Nano Letters* 24, no. 30 (2024):9391-9398. doi:10.1021/acs.nanolett.4c02658

20. T. Zhao, W. Yue, Q. Deng, et al., “Neuromorphic Transistors Integrating Photo‐Sensor Optical Memory and Visual Synapses,” *Advanced Materials* 37, no. 27 (2025): 2419208. doi:10.1002/adma.202419208

21. B. Alqahtani, H. Li, A. Syed, et al., “From light sensing to adaptive learning: hafnium diselenide reconfigurable memcapacitive devices in neuromorphic computing,” *Light: Science & Applications* 14, no. 1 (2025): 30. doi:10.1038/s41377-024-01698-6

22. J. Woo, D. Lee, Y. Koo, et al., “Dual functionality of threshold and multilevel resistive switching characteristics in nanoscale HfO_2_-based RRAM devices for artificial neuron and synapse elements,” *Microelectronic Engineering* 182 (2017):42-45. doi:10.1016/j.mee.2017.09.001
